# Separation of Volatile Organic Compounds in TAMOF-1

**DOI:** 10.1021/acsami.2c05223

**Published:** 2022-07-01

**Authors:** Carmen González-Galán, Mabel de Fez-Febré, Stefano Giancola, Jesús González-Cobos, Anton Vidal-Ferran, José Ramón Galán-Mascarós, Salvador R. G. Balestra, Sofía Calero

**Affiliations:** † Department of Physical, Chemical, and Natural Systems, Universidad Pablo de Olavide, Ctra. Utrera Km 1, ES-41013 Seville, Spain; ‡ Institute of Chemical Research of Catalonia (ICIQ), The Barcelona Institute of Science and Technology (BIST), Av. Països Catalans 16, ES-43007 Tarragona, Spain; § Departament de Química Física I Inorgànica, Universitat Rovira i Virgili, Marcel. Lí Domingo 1, 43007 Tarragona, Spain; ∥ Catalan Institution for Research and Advanced Studies (ICREA), Passeig Lluis Companys 23, ES-08010 Barcelona, Spain; ⊥ Instituto de Ciencia de Materiales de Madrid, Consejo Superior de Investigaciones Científicas (ICMM-CSIC), c/ Sor Juana Inés de La Cruz, 3, 28049 Madrid, Spain; # Materials Simulation and Modelling, Department of Applied Physics, Eindhoven University of Technology, 5600 MB Eindhoven, The Netherlands; ¶ Department of Inorganic and Organic Chemistry, University of Barcelona, C. Martí i Franquès 1-11, 08028 Barcelona, Spain

**Keywords:** TAMOF-1, VOCs, xylene, hexane, benzene, cyclohexane, separation

## Abstract

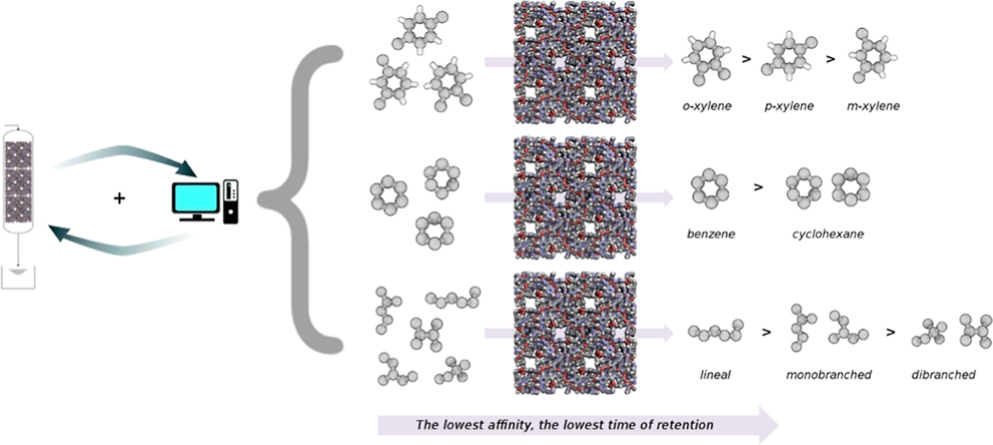

Separation of volatile organic compounds is one of the most studied processes in industry. TAMOF-1 is a homochiral metal–organic framework with a crystalline network of interconnected ≈1 nm channels and has high thermal and chemical stability. Thanks to these features, it can resolve racemic mixtures of chiral drugs as a chiral stationary phase in chromatography. Interestingly, the particular shape and size of its channels, along with the presence of metallic centers and functional groups, allow establishing weak but significant interactions with guest molecules. This opens interesting possibilities not only to resolve racemates but also to separate other organic mixtures, such as saturated/unsaturated and/or linear/branched molecules. In search of these applications, we have studied the separation of volatile organic compounds in TAMOF-1. Monte Carlo simulations in the grand-canonical ensemble have been carried out to evaluate the separation of the selected molecules. Our results predict that TAMOF-1 is able to separate xylene isomers, hexane isomers, and benzene–cyclohexane mixtures. Experimental breakthrough analysis in the gas phase and also in the liquid phase confirms these predictions. Beds of TAMOF-1 are able to recognize the substitution in xylenes and the branching in hexanes, yielding excellent separation and reproducibility, thanks to the chemical and mechanical features of this material.

## Introduction

Metal–organic frameworks (MOFs) are a class of porous crystalline materials which are made by the coordination of organic ligands and metallic centers, producing an extended network in the solid state. Due to their versatility, the applications described for MOFs are very extensive and include catalysis, separation and purification, gas storage, and drug delivery, among others.^1−4^ Focusing on the separation processes, the ability to implement pores of specific sizes and shapes offers the possibility of synthesizing new materials for selective separation. An example is the design of MOFs for selective adsorption of gases, hydrocarbons, aromatic compounds, or enantiomers.^5−10^

TAMOF-1 (triazole acid metal–organic framework) is a chiral MOF with high stability upon water and organic solvents and permanent porosity. It is formed by the coordination of copper(II) (as a metallic center) and (*S*)-3-(1*H*-imidazol-5-yl)-2-(4*H*-1,2,4-triazol-4-yl)-propanoic acid (as an organic linker) (Figure S1). Due to the presence of stereogenic centers in their linkers, TAMOF-1 can be used for the separation of certain chiral molecules. Recently, we have reported the separation of model racemic mixtures (including drugs) using a bed of TAMOF-1 incorporated into HPLC columns.^10^ Moreover, the reported mechanism of separation by Corella-Ochoa et al., that is, a combination of weak host–guest interactions and weak preferential adsorption between different compounds could make TAMOF-1 extensible as a versatile molecular sieve to a wide range of non-chiral molecules. The type of tortuous, narrow, persistent (chiral), 3D-connected channels, together with the designed column device, seems to indicate that a small difference in the adsorption properties would imply not only a preferential attachment mechanism toward enantiomer selectivity but also the transport of the whole through the column. In this work, we assume that this mechanism will also be efficient for structural isomers of compatible size with the porosity of TAMOF-1; for instance, the separation of saturated/unsaturated compounds based on the interaction with the metal or the separation of linear/branched molecules based on the shape of the channels. Separation of volatile organic compounds, one of the most studied processes in industry, includes all the earlier mentioned groups.

Aromatic hydrocarbons are a relevant fraction of volatile organic compounds (VOCs). Among them, xylene is an important environmental pollutant. The three isomers vaporize and divide into other harmless chemicals. Short-term exposure to xylene isomers is related to some health problems such as irritation of the nose or eye and other neurological, reproductive, or gastrointestinal toxic effects. Long-term exposure could cause hazardous effects on several human systems: respiratory, central nervous, cardiovascular, and renal systems.^11^ The capture and separation of xylene isomers (*o*-, *m*-, and *p*-xylene) is a complex process because of the similarities of the three molecules. In fact, the only difference is the relative position of the two CH_3_ groups. Although their properties are very similar, the applications differ for each isomer.^12^*p*-Xylene is essential for synthesizing polyethylene terephthalate and polybutylene terephthalate. *o*-Xylene and *m*-xylene are raw materials for the production of phthalic anhydride and isophthalic acid, respectively. *p*-Xylene is the most widely used among the three isomers in industry and thus its separation is important. Regarding conventional methods, distillation is only useful for separating *o*-xylene due to the difference between their boiling points (*o*-xylene: 417.5 K, *m*-xylene: 412.3 K, and *p*-xylene: 411.5 K).^13^ More recently, porous materials (including MOFs^14−16^ and zeolites^17−19^) have also been used for this purpose, showing different selectivities toward the isomers depending on the framework selected. Castillo et al.^14^ studied the separation of xylene isomers using MIL-47 via Monte Carlo simulations finding adsorption selectivity (*o*- → *p*- → *m*-xylene) due to the specific interactions related to the CH_3_ groups. Peralta et al.^15^ carried out the targeted separation using ZIF-8 based on the flexibility of the selected structure, obtaining a great separation in the gas phase. Jin et al.^16^ used a novel microporous material as the molecular sieve to separate *p*-xylene from a mixture of the three isomers. Among these isomers, separation of *m*-xylene using zeolites has also been reported. In particular, Yuan et al.^20^ studied the separation of xylene isomers using MFI-type zeolite membranes. They found lower diffusion for *m*-xylene than for the other isomers. As has been commented above, Rasouli et al.^18^ also separated *m*-xylene from a mixture of the three isomers using NaY zeolite. They investigated the effect of different factors during the separation, finding that the nanoscale increases the selectivity.

Benzene–cyclohexane separation is one of the most complex process in industry due to similarities between these two molecules. For instance, the difference in their boiling points is only 0.6 K, and this makes conventional methods such as distillation inefficient to carry out the separation. In search for new alternatives, many studies related to targeted separation using porous materials have been reported.^21−25^ Zeolites, MOFs, and derived materials have been used to separate these two molecules based on the π-complexation (also named π-bonding). Mukherjee et al.^23^ carried out the separation using some members of the MOF-74 family due to the interaction between the aromatic ring and the open metal site. Liu et al.^21^ explained the separation based on the same criterion and attributed the lower diffusion of benzene to this phenomenon. π-Complexation is related to the interaction of the aromatic ring (benzene) and the metallic center (MOFs) or the cation (zeolites or aluminosilicates), and it has also been described in other systems.^26,27^ Separation of benzene from a mixture of it with cyclohexane is important due to the toxicity of the first one.^28−30^

Separation of isomers of hexane is an important process in industry due to its relation to the isomerization of alkanes. This process is gaining increasing importance in the petroleum industry.^31,32^ In a conventional reactor for hexane isomerization, the product contains a distribution of chemical compounds including the unreacted *n*-hexane, its monobranched isomers (2-methylpentane and 3-methylpentane), and its dibranched isomers (2,2-dimethylbutane and 2,3-dimethylbutane). Commonly, LTA zeolite is used as a molecular sieve, allowing the adsorption and diffusion of linear molecules and recycling *n*-hexane as a chemical precursor of the isomerization reactor. The degree of branching is related to the octane number, and dibranched isomers are preferred.^33^ Considering this fact, separation of hexane isomers based on the degree of branching is required. Many studies about hexane isomer separation using porous materials are found in the literature.^34−38^ Mendes et al.^35^ used ZIF-8 to carry out the separation of hexane isomers, obtaining high selectivity toward *n*-hexane. Ferreira et al.^38^ used MFI-type zeolites to study the effect of the Al content on the separation of hexane isomers and found that Silicalite-1 is promising for this purpose. Ferreira et al.^39^ also studied the separation of hexane isomers using MFI zeolite and obtained great selectivity toward the less branched isomers (linear > monobranched > dibranched). Separation of hexane isomers is also important due to the possible toxic effects.^40,41^

In this work, we carried out Monte Carlo simulations to study the separation of xylene isomers, hexane isomers, and benzene–cyclohexane using an interesting water-proof MOF with high stability. The shape and size of its channels and the presence of metallic centers offer the possibility to separate linear/branched and saturated/unsaturated molecules. All our computational predictions have been confirmed by experimental data. The corresponding breakthrough curves (BCs) for the mixtures using TAMOF-1 demonstrate its molecular recognition features: TAMOF-1 can distinguish between cyclic compounds based on the interaction with the metal (benzene–cyclohexane system), the degree of branching (hexane isomers), and positional isomers (xylene isomers). In addition to the already reported recognition features in the separation of chiral compounds, TAMOF-1 appears as a unique, outstanding chromatographic stationary phase for molecular separation in the liquid phase and also in the gas phase.

## Experimental Details

### Chemicals and Materials

TAMOF-1, with the molecular formula [Cu(H_2_O)_2_(C_8_H_8_N_5_O_2_)_2_]·6H_2_O, was synthesized following the procedure described in a previous publication.^10^Table 1 shows a summary of crystal data and physical properties of TAMOF-1. Furthermore, complete crystal structure characterization can be found in the aforementioned publication. All chemicals employed were of commercial grade and used without further purification. Xylene isomers: *p*-xylene (C_6_H_4_(CH_3_)_2_, 99%), *m*-xylene (C_6_H_4_(CH_3_)_2_, 99%), *o*-xylene (C_6_H_4_(CH_3_)_2_, 97%); hexane (C_6_H_14_, 99%); 3-methylpentane (C_6_H_14_, 98%); 2-methylpentane (C_6_H_14_, 98%); 2,3-dimethylbutane (C_6_H_14_, 99%); 2,2- dimethylbutane (C_6_H_14_, 99%); cyclohexane (C_6_H_12_, 99%); and benzene (C_6_H_6_, 99.9%) were supplied by Sigma-Aldrich.

**Table 1:** Summary of Crystal Data and Physical Properties of TAMOF-1

formula	[Cu(H_2_O)_2_(C_8_H_8_N_5_O_2_)_2_]·6H_2_O
molecular weight, g/mol	583.93
crystal system	cubic
space group	P4_3_/32
surface area BET, m^2^/g	980.50
pore volume, cm^3^/g	0.38
framework density, g/m^3^	1.14
particle size, μm	0.2–10.0
crystal size, mm^3^	1.5 0.05 × 0.03

### Dynamic Fixed-Bed Column Adsorption Experiments

**Figure 1:**
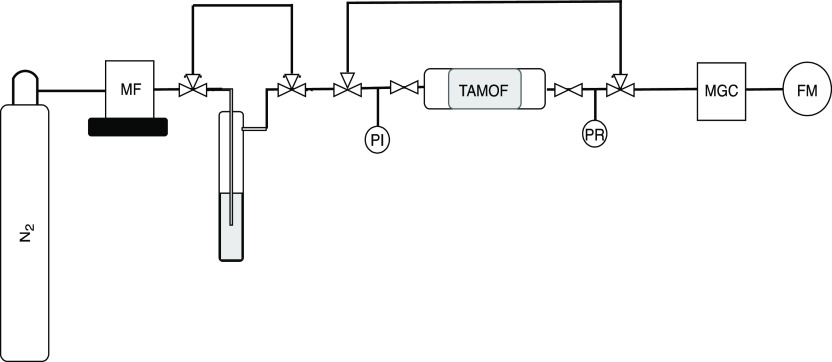
Scheme of the fixed-bed adsorption setup. MF: mass flow controller, PI: pressure indicator, PR: pressure regulator, MGC: micro-gas chromatograph, FM: flow meter.

Fixed-bed adsorption experiments were performed by using the experimental setup shown in Figure 1. Nitrogen (Praxair, 99.999%) was used to vaporize the liquid studied compounds inside a trap (see later). The flows of the inlet N_2_ gas streams were controlled by a set of calibrated mass flow controllers (Bronkhorst EL-FLOW).

**Figure 2:**
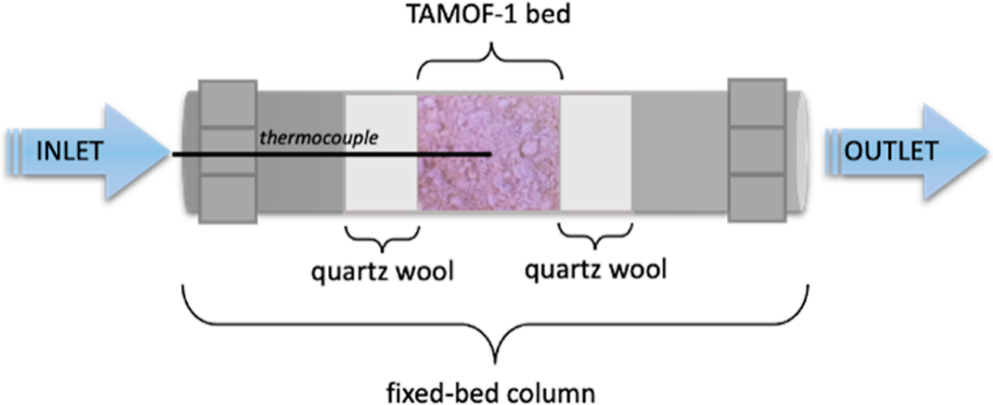
Drawing of the fixed-bed column filled with TAMOF-1.

A manometer and a backpressure controller (Bronkhorst, EL-PRESS) were placed downstream the separation module. A manometer was also used upstream the bed to monitor the actual pressure in the bed under no pressure control conditions. Unless otherwise stated, all pressure values are reported in units of absolute bar (bar). The separation module was heated by a linear power silicone heating wire (*Ø* 3 mm FOR-FLEX NORMAL, Electricfor) rolled around the column; the temperature was measured with a K-type thermocouple (Thermocoax) inserted in the middle of the bed, and it was controlled with a temperature controller EZ-Zone (Watlow). The outlet stream of the column was on-line analyzed using a micro gas chromatograph (MGC, Agilent MicroGC 490) equipped with Molsieve MS5A, using Ar as a carrier gas (99.999% purity), and a PoraPLOT U column, using He as the carrier gas (99.999% purity), along with thermal conductivity detectors. A separation module with 50 mm length and 10 mm inner diameter was employed as an MOF container. In particular, the as-synthesized TAMOF-1 powder (700 mg) was packed in the middle of this module to form a bed with 10 mm length and 10 mm diameter, and the rest of the volume was filled with glass wool, as shown in Figure 2.

**Figure 3:**
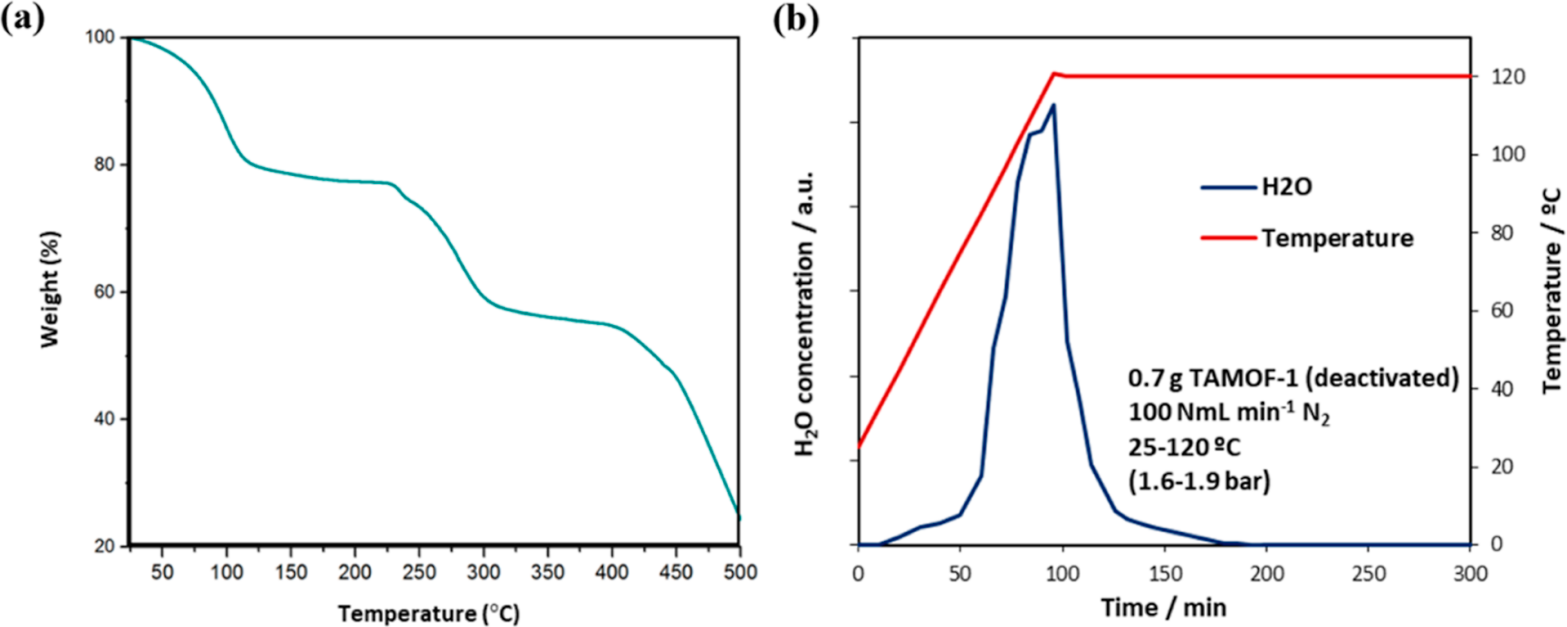
(a)TGA of TAMOF-1 (as-synthesized). (b) In situ monitoring of TAMOF-1 activation in a fixed-bed column under N_2_ flow stream by water analysis in the effluent with a microgas chromatograph.

TAMOF-1 thermal stability up to 423–433 K was previously confirmed by thermogravimetric analysis (TGA) using a TGA/SDTA851 Mettler (Figure 3a). The initial weight loss of 22% is attributed to TAMOF-1 dehydration. Prior to gas separation measurements, TAMOF-1 was activated in situ for water removal at 393 K (1 K min^–1^) using N_2_ stream (100 NmL min^–1^). Activation was in situ monitored by MGC analysis of the water content in the outlet stream (see Figure 3b). One can observe that TAMOF-1 is totally activated after 3 h following this activation procedure, which could be accelerated by increasing either the N_2_ flow or the heating rate.

To perform fixed-bed column adsorption experiments, nitrogen-vaporized compounds were produced by the nitrogen bubbling method. The reservoirs, filled with the liquid compounds, were conditioned to control the vapor pressure. Nitrogen was passed through the container at a desired rate. A continuous flow of vaporized components was obtained and subsequently directed toward the packed column where the adsorption takes place. The partial pressure of each compound was calculated with the Wagner equation^5^ at the given temperature to obtain the approximate concentration. Unless otherwise specified, after the experiments, the MOF was cleaned with N_2_ sweep gas for 8 h (at least) at a total flow rate of 100 NmL min^–1^, 393 K of temperature, and 1.2 bar of pressure to ensure the total desorption of any gas trace from the MOF before the subsequent experiment. The effect of operation conditions on the desorption experiments has also been evaluated.

Experimental BCs^2,3^ are typically plotted as the gas concentration fractions, denoted as *C*/*C*_0_, where *C* and *C*_0_ are the outlet and inlet concentrations, respectively, as a function of time. It is important to mention that before saturation, a roll-up effect takes place, which is very common in binary mixture breakthroughs. This consists of the displacement of part of the adsorbed amount of the first-eluting gas compound by the second-eluting one when the delayed mass front of the latter faces the bed full of the former.^4^ This leads to the elution, for a short period of time, of the first-eluting gas flow rate higher than that in the feeding stream. To plot the BCs, the corresponding transit time or death time of the gas, which is the time necessary to the gas mixture to flow through the separation module and all the pipes leading to the analyzer, has been calculated and subtracted at each operation condition.

In order to evaluate the separation properties, different parameters were calculated from the BC. The breakthrough time has been defined as the time it takes for the gas to reach 1% of the respective inlet concentration at the adsorption column outlet. The adsorption capacity at saturation point (*q*_s_) for each gas compound, in mmol g^–1^, is the most common adsorption capacity parameter found in breakthrough studies and refers to the total amount of a gas component adsorbed on the MOF before this gets saturated, that is, when the gas reaches the inlet concentration at the outlet stream. It can be estimated from the integration of BCs through the following equation
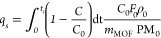
1where *t*_*s*_, in min, is the saturation time or equilibrium time, defined as the time to reach 99% of the inlet concentration in the outlet stream; *C* and *C*_0_ are the outlet and inlet gas concentrations, respectively; *F*_0_ is the overall inlet molar flow rate, in mL/min; *m*_MOF_ is the mass of the MOF (activated) in units of *g*; ρ_0_ is the density of feed gas in units of g/L; and PM_0_ is the molecular weight of the gas in units of g mol^–1^. The adsorption selectivity at saturation (*S*_s_) is estimated at the given pressure and temperature conditions through the following equation
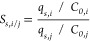
2where *q*_*s,i*_ and *q*_*s,*j_ are the individual adsorption capacities of gases measured after MOF saturation. *S*_*s*,*i*/*j*_ ≥ 1.1 means that the mixture can be successfully resolved by the adsorbent via chromatography. *S*_*s*,*i*/*j*_ > 1.5 indicates excellent separation features.

## Simulation Details

Simulations were performed using the RASPA code.^42^ TAMOF-1 was considered a rigid framework, and the charges were taken from a previous work using Qeq calculations.^43^ The Lennard-Jones parameters were taken from Universal Force Field for metallic centers and DREIDING force field for organic linkers.^44,45^ Host–guest and guest–guest interactions were described using Lorentz–Berthelot mixing rules. This procedure has been extensively validated.^46−49^ A rigid model was also used for xylene isomers and the benzene–cyclohexane system. Note that *chair* and *twist-boat* (*tboat*) conformers were selected for cyclohexane. For hexane isomers, a flexible model was considered, and parameters were taken from Transferable Potentials for the Phase Equilibria force field.^50^ All molecules were described using united-atoms. Intramolecular potential included bond, bend, dihedral torsion, and electrostatic Coulomb terms.

Heat of adsorption was calculated for all the molecules using the Widom Test Particle Insertion method at infinite dilution.^51^ To insert the molecules inside the framework, the Configuration Bias Monte Carlo method was used. Monte Carlo simulations in the grand-canonical ensemble (μ*VT* MC) were carried out to obtain the single adsorption isotherms of the guest molecules at different values of temperature (depending on the adsorbate). This ensemble allows the number of molecules to fluctuate by fixing the chemical potential (μ), the volume (*V*), and the temperature (*T*).

We have calculated binding energies and geometries for some of the components of the mixtures to show what kinds of host–guest interactions are relevant. We have obtained these geometries by performing energy minimizations. To avoid obtaining structures in local minima, we performed a previous *NVT* MC, from which we chose a snapshot of the trajectory with the energy minimum. We select the coordinates of this snapshot to perform the energy optimizations.

Ideal Adsorbed Solution Theory (IAST)^52^ implemented with the GAIAST code^53^ was used to predict the mixture adsorption based on the behavior of the single adsorption isotherms of the pure components. The Langmuir–Freundlich dual-site model was used for all systems. The adsorption mechanism on TAMOF-1 detailed in the Introduction section allows us to hypothesize that the adsorption and separation processes can be studied with IAST calculations as the system fulfils all the prescriptions for its use.^54^ We have also performed μ*VT* MC simulations for the multi-component adsorption isotherms to validate some of the IAST predictions (see Figure S2).

The average occupation profiles were computed using a homemade code by averaging the entire trajectory recorded during grand-canonical Monte Carlo simulations at a selected value of pressure. The π-interaction between molecules—xylene isomers and benzene—was also computed by using a homemade code. We have considered all the parallel and perpendicular aromatic stacking arrangements.^55^ For both arrangements, we used a geometrical criterion based on two cutoffs: (i) the distance between the two aromatic rings and (ii) the angle between the aromatic rings that should be between 0 and 30° or 60 and 120° for parallel and perpendicular arrangements, respectively.^56^ To obtain the mentioned distances, we have used the first minimum of the radial distribution function (RDF) from molecular dynamics simulations of the molecules in the bulk (reproducing the density).

## Results

### Separation of Xylene Isomers

**Figure 4:**
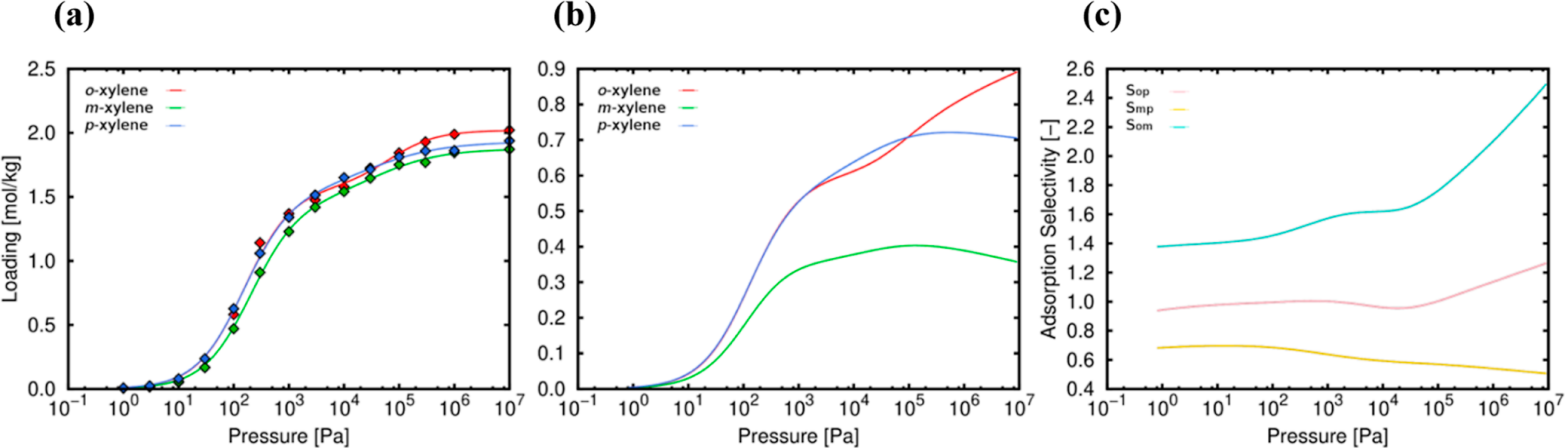
(a) Single adsorption of *o*-xylene (red), *m*-xylene (green), and *p*-xylene (blue); (b) equimolar mixture isotherms (same code color); and (c) adsorption selectivity of *S*_op_ (pink line), *S*_mp_ (yellow line), and *S*_om_ (light-blue line) in TAMOF-1 at 393 K. Lines and symbols are related to simulations and IAST prediction, respectively.

To study the separation of *o*-xylene, *m*-xylene, and *p*-xylene, single adsorption isotherms of the three isomers have been obtained at 393 K using Monte Carlo simulations in the grand-canonical ensemble. The GAIAST code was used to obtain multi-component adsorption isotherms. We have also calculated the adsorption selectivities to show the separation of the isomer from the other two isomers. Figure 4 shows the single adsorption isotherms, the IAST prediction for the mixture, and the adsorption selectivity at 393 K. As shown in the adsorption isotherms of the pure components, the initial pressure at which the molecules start to adsorb is almost the same for the three compounds. The calculated heat of adsorption of the isomers is also very similar (*o*-xylene: −54.94 kJ/mol, *m*-xylene: −55.84 kJ/mol, and *p*-xylene: −56.66 kJ/mol). This means that the interaction between the three molecules and the framework is alike at infinite dilution.

Multi-component adsorption isotherms show that *o*-xylene and *p*-xylene are preferred over *m*-xylene. This can also be observed in the adsorption selectivity (*S*_*ij*_*, i,j* = *o, m, p*). As shown in Figure 4, *S*_op_ has a value of about 1, showing a similar trend in the adsorption of *o*-xylene and *p*-xylene (pink line). *S*_mp_ (yellow line) and *S*_om_ (blue line) are also related to the preference for *o*-xylene and *p*-xylene over the *m*-isomer, respectively. To explain the selectivity during the separation, the interaction between the molecules of each isomer inside the framework has been analyzed. First, we focused on the π-interaction (*n*_π_) considering the aromatic rings in all the molecules. As shown in Figure S3, in the Supporting Information, the interaction increases with the adsorption loading due to the presence of a higher number of molecules inside the framework. However, this π-interaction is very similar for all cases, indicating that it is not a key factor in the separation of xylene isomers.

**Figure 5:**
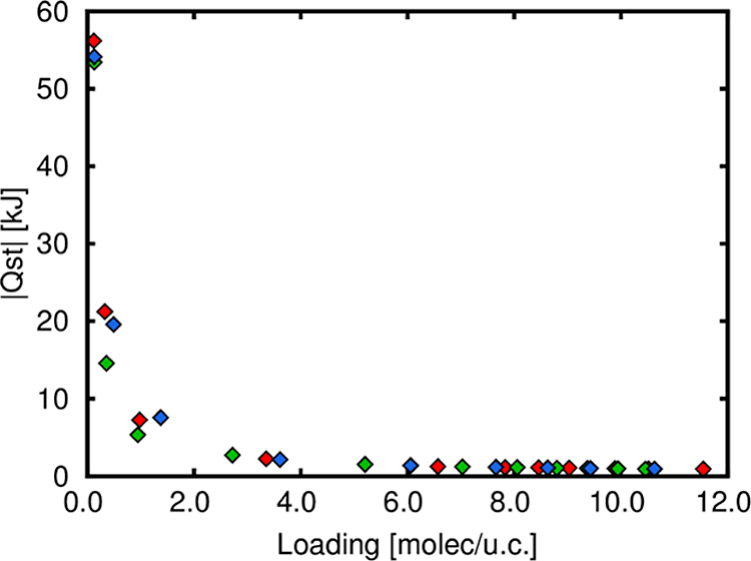
Heat of adsorption per molecule of *o*-xylene (red), *m*-xylene (green), and *p*-xylene (blue) in TAMOF-1 at 393 K.

The selectivity in favor of *o*-xylene and *p*-xylene could be related to the interaction with the framework and in particular to the characteristic shape of the channels in TAMOF-1. As a result, the heats of adsorption of the three isomers were calculated as a function of loading (Figure 5). As can be seen, the physical basis behind the separation is based on the concept of “preferential attachment”: the small difference in adsorption enthalpies does not seem to be sufficient to generate separation at the maximum dilution limit. However, the entry of a first isomer type generates a break in the symmetry of which type of molecule enters next, which is linearly determined by the heat of adsorption with *n* molecules already adsorbed (Figure 5), that is, the adsorption of a first molecule of type “*a* = *o*-, *m*-, or *p*-” generates a more affine environment for a second molecule of the same species. The narrow channel favors this behavior, and, in turn, the 3D porosity enables the transport of less trapped molecules.

**Figure 6:**
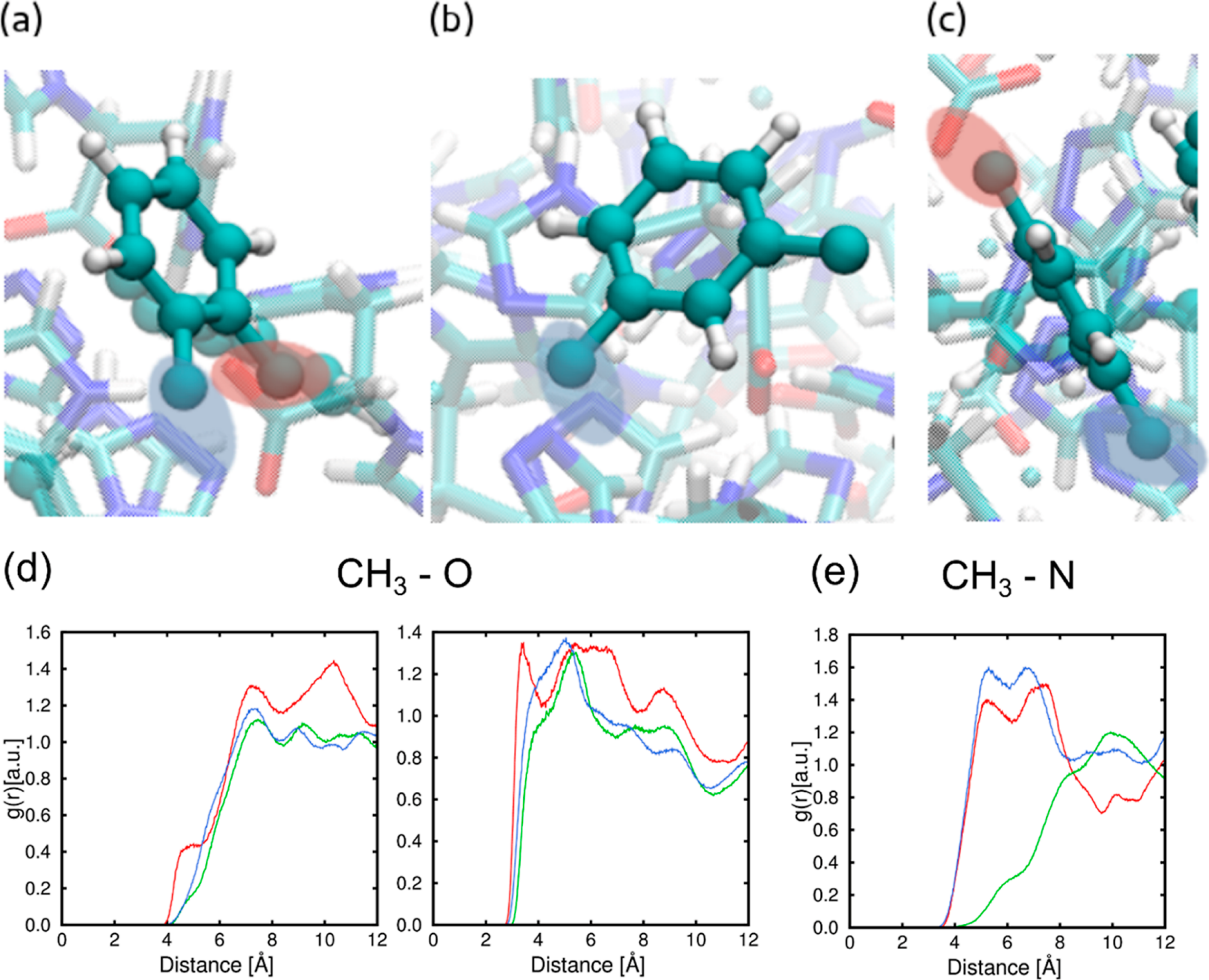
(Top) Snapshot of the binding geometry of (a) *o*-xylene, (b) *m*-xylene, and (c) *p*-xylene in TAMOF-1. The most significant interactions are highlighted in each snapshot. (Bottom) RDFs between the CH_3_ functional groups of xylene and (d) O atoms of the carboxylate group of the structure and (e) N atoms of the heterocycle with two nitrogen atoms. The code color is red, green, and blue for *o-*, *m-*, and *p-*xylene, respectively.

To identify the reason of the strongest interaction of *o*-xylene and *p*-xylene with the framework at infinite dilution, we calculate the binding geometries for each isomer, and we extract the RDFs of the relevant interactions from MC simulations as well. Figure 6a–c shows the binding geometries for each isomer in TAMOF-1. As can be observed from the shaded circles, *o*-xylene and *p*-xylene interact with the two CH_3_ groups with the closest ligands (O and N atoms), while the interaction of *m*-xylene is with one CH_3_ group and the closest ligand (N atoms); however, it does not compete with the interactions of the other two isomers, that is, the distances between the isomers and neighboring ligands are shorter for *o*-xylene and *p*-xylene than for *m*-xylene. This is clearly visible with the study of the RDFs, which are visible in the ESI (see Figures S11 and S10). An extract of that information is shown in Figure 6d,e. *o-*Xylene interacts mainly via the CH_3_ groups with the oxygen atoms of the carboxylate groups in the structure. *o-* and *p-*xylenes compete in the interaction with the nitrogen atoms of the structure, mainly those related to the heterocycle with two nitrogen atoms (N_4_ and N_5_ atoms, see Figure S11 for more details).

**Figure 7:**
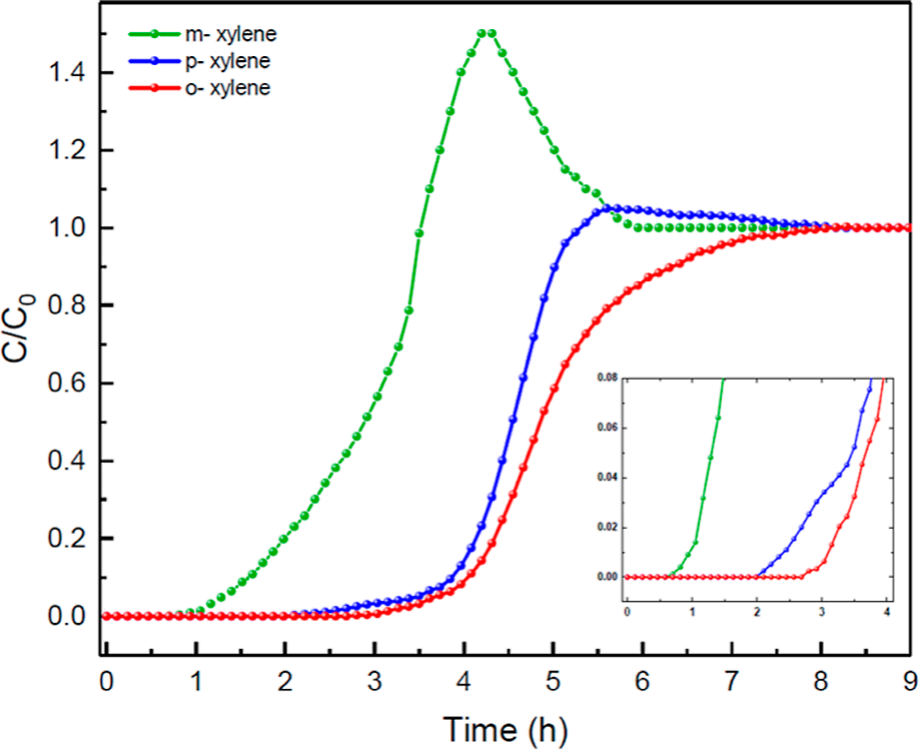
Experimental BCs for an equimolar gas mixture of xylene isomers: *m*- (*green*), *p*- (*blue*), and *o*-xylene (*red*) through a TAMOF-1 bed (0.7 g) at a total flow rate of 30 NmL min^–1^, 1.2 bar, and 393 K.

To validate the model and force field parameters used in the molecular simulations, we carried out experimental separations using columns packed with TAMOF-1. Figure 7 shows the BCs for an equimolecular gas mixture of xylene isomers (*o*- → *m*- → *p*-xylene) at atmospheric pressure and *T* = 393 K through a bed of TAMOF-1. Typical S-shaped BCs were found with the elution order of *m*- > *p*- > *o*-xylene. The corresponding adsorption capacity values are 0.011^—^, 0.016, and 0.017 mmol g^–1^, respectively. The selectivity for *o*-xylene exhibits relatively good resolution with the other two isomers, with *S*_o-/m-_ = 1.54 and *S*_o-/p-_ = 1.06, compared with the best resolutions reported for this mixture for other adsorbents, for example, *S*_o-/p-_ = 6.7 and *S*_o-/m-_ = 10.0, and an equal elution sequence for MAF-6^57^ or *S*_o-/p-_ = 6.33 but no resolution for *o-/m-*xylenes for HKUST-1^58^ or *S*_o-/m-_ = 1.84 but no resolution for *o-/p-*xylenes for MIL-101(Cr) and *S*_o-/p-_ = 3 and no resolution *o-/m-*xylenes for MIL-53(Al).^59,60^ The most general commercial nonpolar capillary column, HP-5MS, does not exhibit separation at all.^57^ The fact that *p-*xylene elutes after *m-*xylene runs counter to most of the column processes—for example, UIO-66 and HKUST-1. This mechanism was detailed by He et al.^57^ for MAF-6, and it is possibly related to the fact that TAMOF-1 as well as MAF-6 or MIL-47 interacts more strongly with the less polar isomer, *p-*xylene.

### Separation of Hexane Isomers

**Figure 8:**
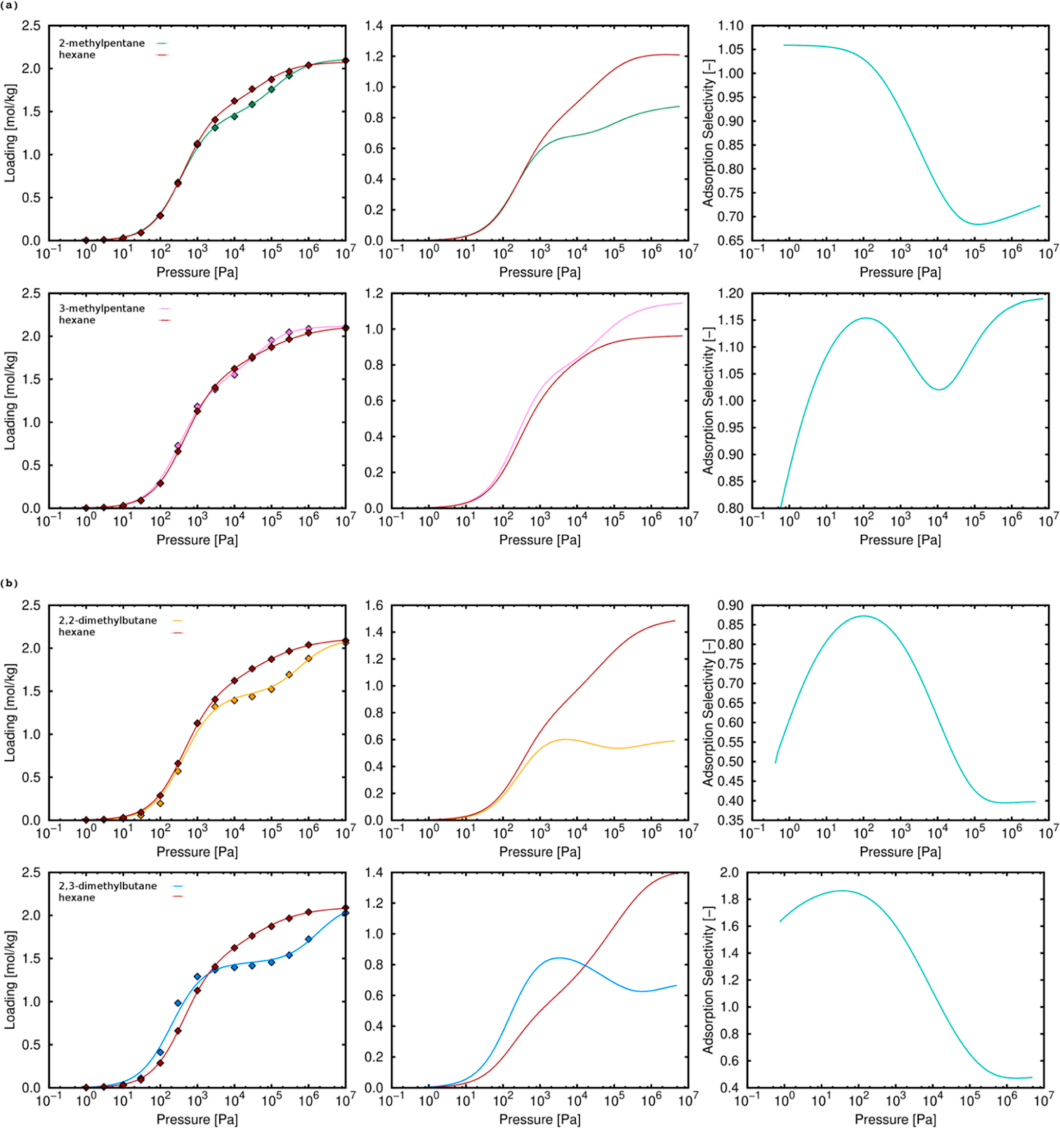
Single adsorption, equimolar mixture isotherms, and adsorption selectivity (C_1_/C_2_, in the same order) of (a) *n*-hexane-monobranched isomers and (b) *n*-hexane-dibranched isomers in TAMOF-1 at 433 K. Lines and symbols are related to the calculated and IAST-predicted isotherms, respectively.

**Figure 9:**
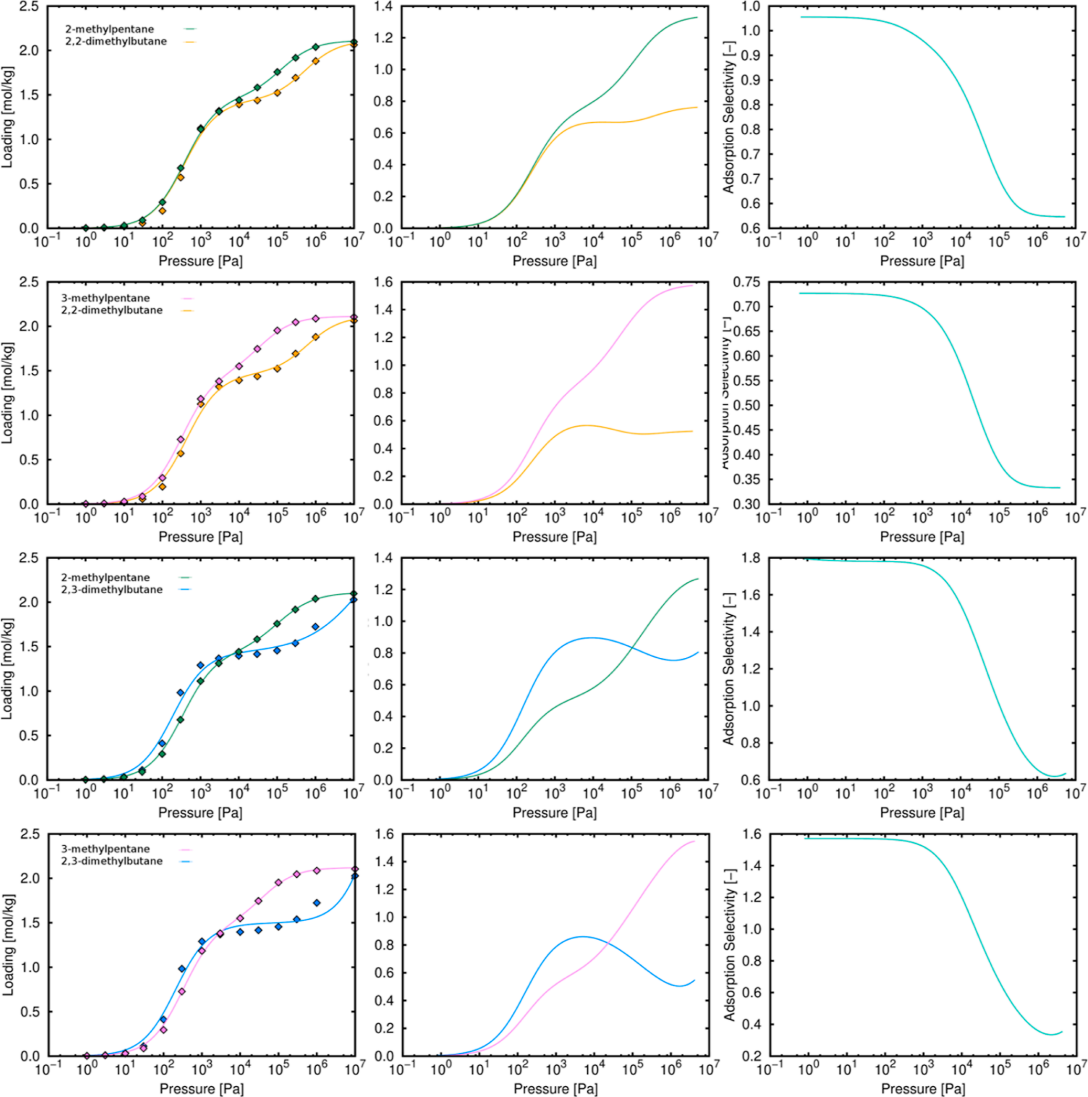
Single adsorption, equimolar mixture isotherms, and adsorption selectivity of monobranched–dibranched isomers in TAMOF-1 at 433 K. Lines and symbols are related to the calculated and IAST predicted isotherms, respectively.

We evaluated the capacity of TAMOF-1 to separate linear, monobranched, and dibranched isomers of hexane isomers. We performed μ*VT* MC simulations to obtain the single adsorption isotherms of all the isomers at 433 K. Multi-component adsorption isotherms were obtained from IAST at the same temperature. The separation results for an equimolar mixture of monobranched isomers and dibranched isomers can be found in the Supporting Information (Figures S4 and S5) and indicate separation between isomers with the same degree of branching. Figure 8 shows the single and multi-component adsorption isotherms and the adsorption selectivity of all the mixtures including (a) linear–monobranched and (b) linear–dibranched isomers. We found that there is a preference for the linear alkane and only for 3-methylpentane, this trend is not present. Figure 9 shows the single and multi-component adsorption isotherms and the adsorption selectivity of all the mixtures including monobranched–dibranched isomers. For all the equimolar mixtures, the monobranched isomer is preferred over the dibranched. The onset pressure is very similar for all isomers, and this is related to the similar values of heat of adsorption obtained for all isomers at infinite dilution (−57.36 kJ/mol for *n*-hexane, −56.92 kJ/mol for 2-methylpentane, −56.70 kJ/mol for 3-methylpentane, −53.93 kJ/mol for 2,2-dimethylpentane, and −56.90 kJ/mol for 2,3-dimethylpentane). Therefore, under these conditions, the interaction with the structure is practically the same in all cases.

To better understand the separation of hexane isomers in TAMOF-1, we now focus on the behavior of each isomer. As shown Figure 8, the linear isomer (*n*-hexane) is preferred over the monobranched and dibranched isomers. Moreover, the selectivity in favor of *n*-hexane in mixtures containing dibranched isomers is higher than that in mixtures containing monobranched isomers. This is related to the preferential adsorption sites for linear and dibranched isomers (see Figure S6). The molecules of *n*-hexane do not exhibit clear preferential adsorption sites (channels and “pockets”), while the dibranched isomers are mainly located in the “pockets”. Linear and monobranched isomers compete for the same adsorption sites, so the separation efficiency is lower than that for dibranched isomers. This behavior is different for 2-methylpentane and 3-methylpentane. It seems that although the adsorption sites are the same, there is a higher occupation of *n*-hexane than that for 2-methylpentane, so *n*-hexane is preferred during adsorption. In the case of the *n*-hexane/3-methylpentane mixture, although the adsorption site is the same for both, there is a higher interaction between 3-methylpentane and the framework, and this isomer is preferred at the end (although the similar behavior causes a small separation in this case) (see Figure S7). The same behavior can be found in the study of the interactions between adsorbents and adsorbates in the RDF (see Figure S12). Note that the most representative interactions have been selected. As can be seen, the interactions between the CH_3_ groups of linear and monobranched isomers are similar, considering N_2_ and N_3_ atoms of the framework (see Figure S11 for the labeling). It agrees with the fact that linear and monobranched isomers compete for the same adsorption sites. In the case of dibranched isomers, differences are observed in all cases. For N_4_ atoms, a higher interaction with the linear isomers is present in the RDF, showing the preferential selectivity toward *n*-hexane.

Considering the observations commented above, comparing monobranched and dibranched isomers, we expected selectivity toward the monobranched isomers. Figure 9 shows that monobranched isomers are preferred over dibranched isomers and this agrees with the separation of linear–dibranched isomers. This is because the occupation profile is very similar for linear and monobranched isomers (see Figure S6). We also found some differences on adsorption between the two monobranched isomers and the two dibranched isomers. For monobranched isomers (Figure S4), there is a preference toward 3-methylpentane and the explanation is the same as for linear–monobranched mixtures. For dibranched isomers (Figure S5), although the two of them have the same adsorption sites, the interaction of 2,3-dimethylbutane is stronger than that of 2,2-dimethylbutane (see Figure S7), so the separation is in favor of 2,3-dimethylbutane.

**Figure 10:**
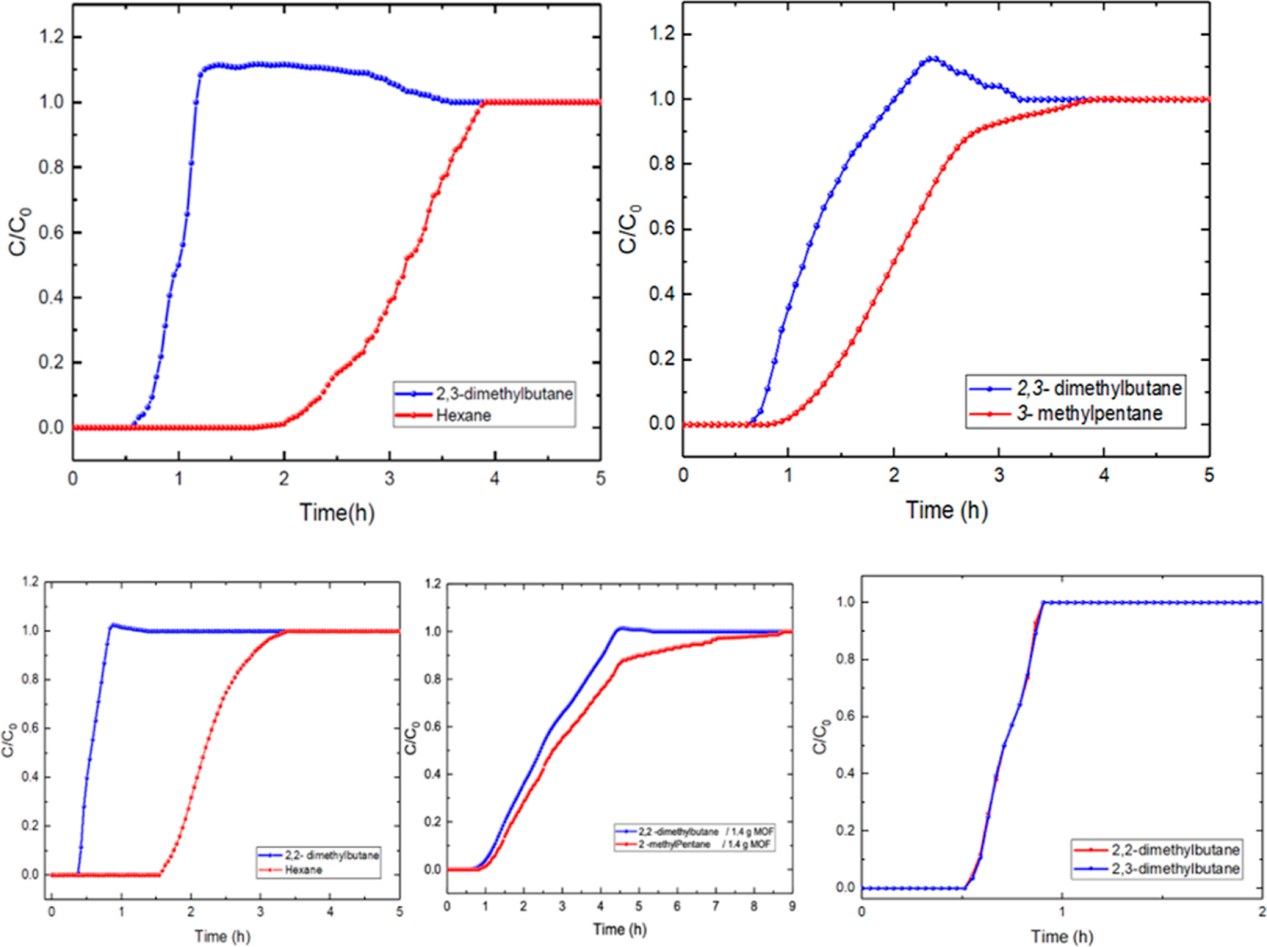
Experimental BCs for a binary gas mixture of *n*-hexane/2,3-dimethylbutane (0.03%/0.04%, v/v) (top-*left*) and 2,3-dimethylbutane/3-methylpentane (0.04%/0.04%, v/v) (top-*right*) through TAMOF-1 (0.7 g) at a total flow rate of 30 NmL min^–1^, 293 K, and 1.2 bar. Experimental BCs for a binary gas mixture of *n*-hexane/2,2-dimethylbutane (0.03%/0.06%, v/v) (bottom-*left*) and 2,2-dimethylbutane/2-methypentane (0.06%/0.04%, v/v) (bottom-*middle*) through a bed of TAMOF-1 (0.7 g, left; 1.4 g, right) at a total flow rate of 30 NmL min^–1^, 293 K, and 1.2 bar. Notice that only in the last case, the amount of MOF was doubled to 1.4 g. Experimental BCs of TAMOF-1 for a binary gas mixture of 2,3-dimethylbutane/2,2-dimethylbutane (0.04%/0.06%, v/v) at a total flow rate of 30 NmL min^–1^, 293 K, and 1.2 bar (bottom-right).

To identify the breakthrough performance for all available hexane isomers, we investigated different binary mixtures. Figure 10 shows the BC curves for the different mixtures in analogous conditions: ambient pressure and 293 K. TAMOF-1 successfully separates these mixtures according exclusively to the degree of branching. Thus, the order of diffusion is dibranched isomers > monobranched isomers > linear hexane. Interestingly, the two dibranched isomers show identical behavior through TAMOF-1 in the gas phase, and the two monobranched isomers also exhibit very similar behavior. The adsorption capacity values are (in mmol g^–1^) *q*_*n*-hexane_ = 0.067, *q*_3-methylpentane_ = 0.046, *q*_2-methylpentane_ = 0.034, and *q*_2,2-dimethylbutane_ = *q*_2,3-dimethylbutane_ = 0.026. In terms of selectivity, the linear hexane is separated in all cases with excellent performance values: *S*_hexane/2,2-dimethylbutane_ = 2.58, *S*_hexane/2,3-dimethylbutane_ = 2.58, *S*_hexane/2-dimethylpentane_ = 1.97, and *S*_hexane/3-dimethylpentane_ = 1.46. Therefore, TAMOF-1 is able to separate hexane isomers showing the following trend in adsorption strength: linear > monobranched > dibranched. This trend is related to the kinetic radius of the isomers: dibranched (ca. 6.2–5.8 Å of kinetic radius), monobranched (ca. 5.0 Å), and linear isomers (ca. 4.3 Å). In the literature, the performance of other porous materials such as MOFs or zeolites has been tested, obtaining different behaviors. On the one hand, zeolite LTA-5A can separate linear and branched isomers, but it is not able to distinguish between monobranched and dibranched isomers. Zeolite beta can separate all the isomers but only partially. On the other hand, MOFs or derivatives that totally separate linear and branched isomers cannot do it taking into account the degree of branching (ZIF-8). MIL-53(Fe) and MAF-6 exhibit the inverse behavior in adsorption strength:^37^ dibranched > monobranched > linear molecules. These results show the versatility for the separation of TAMOF-1 in a wide variety of mixtures.

### Separation of Benzene/Cyclohexane

To study benzene–cyclohexane separation, we calculated the single adsorption isotherms of benzene and cyclohexane at room temperature (298 K) using Monte Carlo simulations in the grand-canonical ensemble. The multi-component adsorption isotherms were obtained using IAST at the same temperature. Note that the total loading of cyclohexane is the sum of both *chair* and *tboat* conformers. The two conformers are equimolar in the reservoir so that the adsorption is based on the affinity or the stability of each of them.

**Figure 11:**
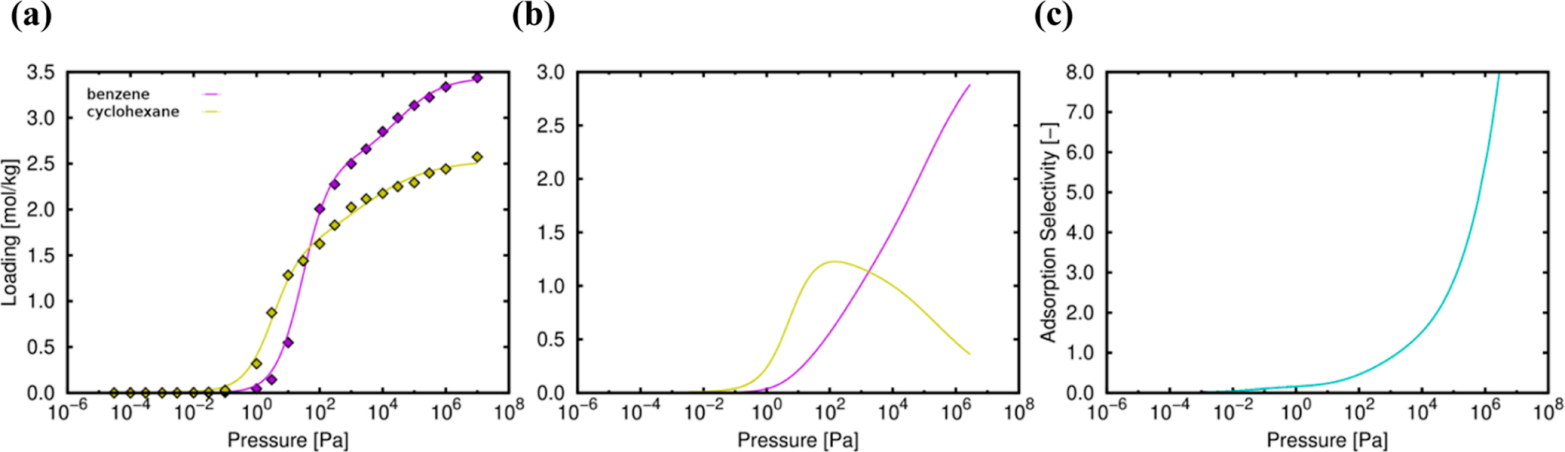
(a) Single-compound adsorption isotherms of benzene (purple line) and cyclohexane (yellow line), (b) equimolar mixture isotherms, and (c) adsorption selectivity *S*_bc_ in TAMOF-1 at 298 K. Lines and symbols are related to the simulated and IAST predicted isotherms, respectively.

The analysis of the single adsorption isotherms (Figure 11) shows that the onset pressure is very similar for both molecules, as expected from the similar values of the heat of adsorption (−42.25 kJ/mol for benzene and −48.54 and −48.47 kJ/mol for *chair* and *tboat* conformers, respectively). These values evidence similar interactions between the framework and the adsorbates (benzene and cyclohexane) at infinite dilution.

**Figure 12:**
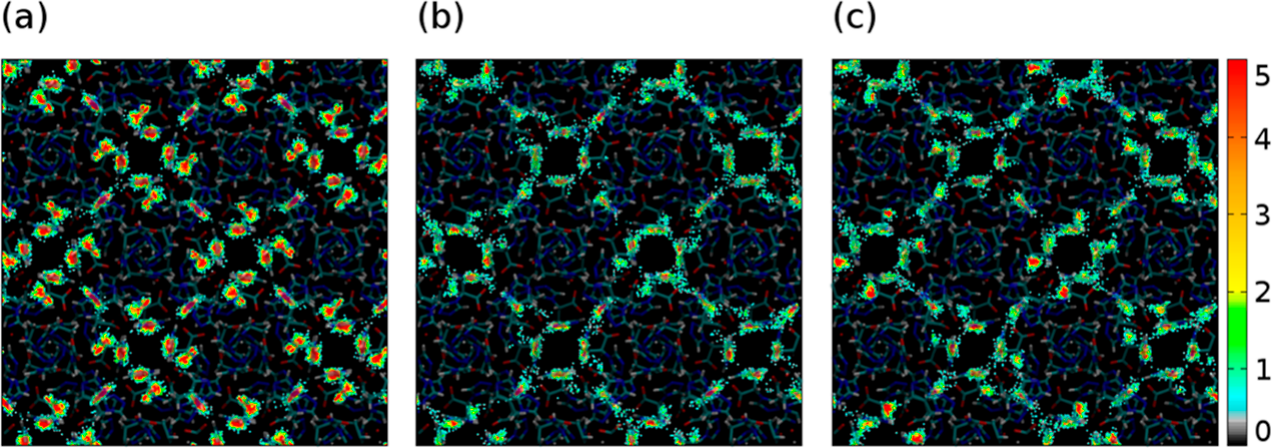
Average occupation profile of (a) benzene, (b) cyclohexane *chair*, and (c) cyclohexane *tboat* at saturation loading.

The adsorption isotherms for the equimolar mixture and the adsorption selectivity (*S*_bc_, blue line) show the final exclusion of cyclohexane in favor of benzene and a clear trend toward the latter (Figure 11). The average occupation profile at saturation pressure clarifies this fact. As shown in Figure 12, benzene is located inside the framework near the metallic centers, the side pocket. Moreover, there is interaction between the molecules of benzene (Figure S8). In the case of cyclohexane, the interaction with the metallic centers does not affect, so all the molecules are located along the channel and the interaction between them is not strong. The represented profile (Figure 12b) corresponds to the *chair* conformation, which is the most stable conformer, but we found the same results for *tboat* (Figure 12c).

**Figure 13:**
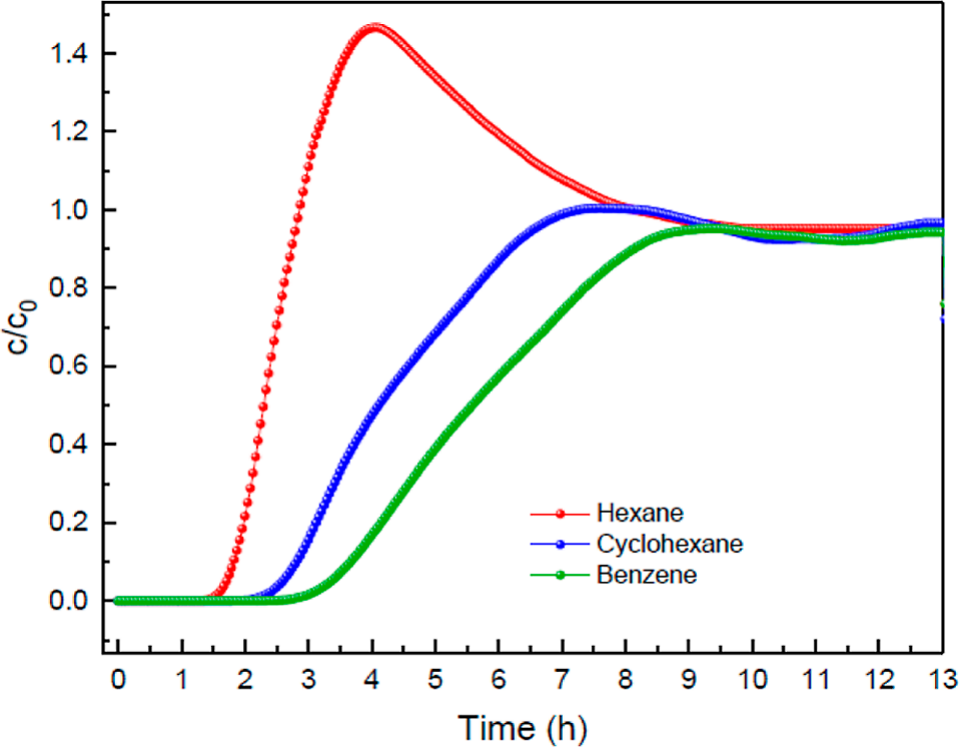
Experimental BCs for a gas mixture of *n*-hexane/cyclohexane/benzene (0.026%/0.016%/0.016%, v/v/v) through a TAMOF-1 bed (0.7 g) at a total flow rate of 30 NmL min^–1^, 298 K, and 1.2 bar.

Figure 13 shows the experimental BCs for a mixture of *n*-hexane, cyclohexane, and benzene through a TAMOF-1 bed. All three compounds are well resolved, with diffusivity in the order of *n*-hexane > cyclohexane > benzene. The adsorption capacities in the corresponding experimental conditions are (in mmol g^–1^) *q*_*n*-hexane_ = 0.046, *q*_cyclohexane_ = 0.057, and *q*_benzene_ = 0.085, with excellent selectivities of *S*_benzene/*n*-hexane_ = 1.85 and *S*_benzene/cyclohexane_ = 1.50. In TAMOF-1, the elution sequence, *n*-hexane > cyclohexane, is opposite to that for UIO-66, Cu3(BTC)2, MIL-47, and MIL-53(Al),^61^ but the retention times are similar, as well as the resolutions.

Therefore, with this mixture, we reverse the tendency of TAMOF-1 to interact more strongly with the more difficult to polarize, as was the case with the xylene isomers. While at low pressures this tendency is confirmed (where cyclohexane interacts strongly with the main channel, as confirmed with the heat of adsorption and with the RDF calculation in Figure S13), as we increase the pressure, benzene starts to enter in the pocket. Thus, we confirm that the main channel is hydrophobic, and the pocket is hydrophilic.

### TAMOF-1 Regeneration and Reusability

**Figure 14:**
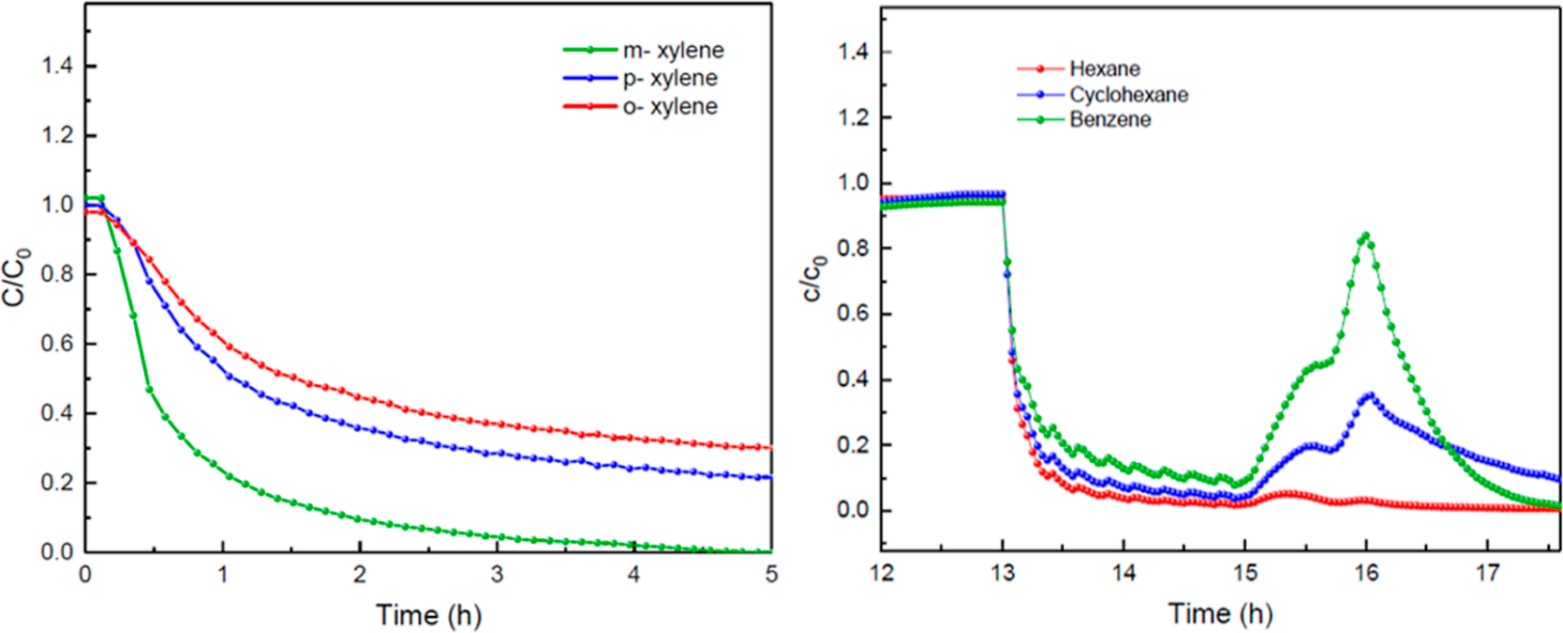
Desorption curves to regenerate the TAMOF-1 bed after the breakthrough experiments of an equimolar gas mixture of xylene isomers *(left)* and a gas mixture of *n*-hexane/cyclohexane/benzene *(right)* at a total N_2_ flow rate of 100 NmL min^–1^, 393 K, and 1.2 bar.

The regeneration and reusability of the stationary phase after column saturation is another important aspect affecting the efficiency of an adsorption/desorption process. A complete, fast, and low-power consumption cleaning step is essential for minimizing operating costs. To evaluate the desorption dynamics for TAMOF-1, we monitored the regeneration step after each breakthrough experiment while passing through the TAMOF-1 bed the pure career gas at the same temperature. Figure 14 (*left* and *right*) shows the desorption steps following the respective adsorption steps shown in Figures 7 and 13, respectively. N_2_ sweep gas was fed through the saturated bed at a total flow rate of 100 NmL min^–1^, 393 K of temperature, and 1.2 bar of pressure.

**Figure 15:**
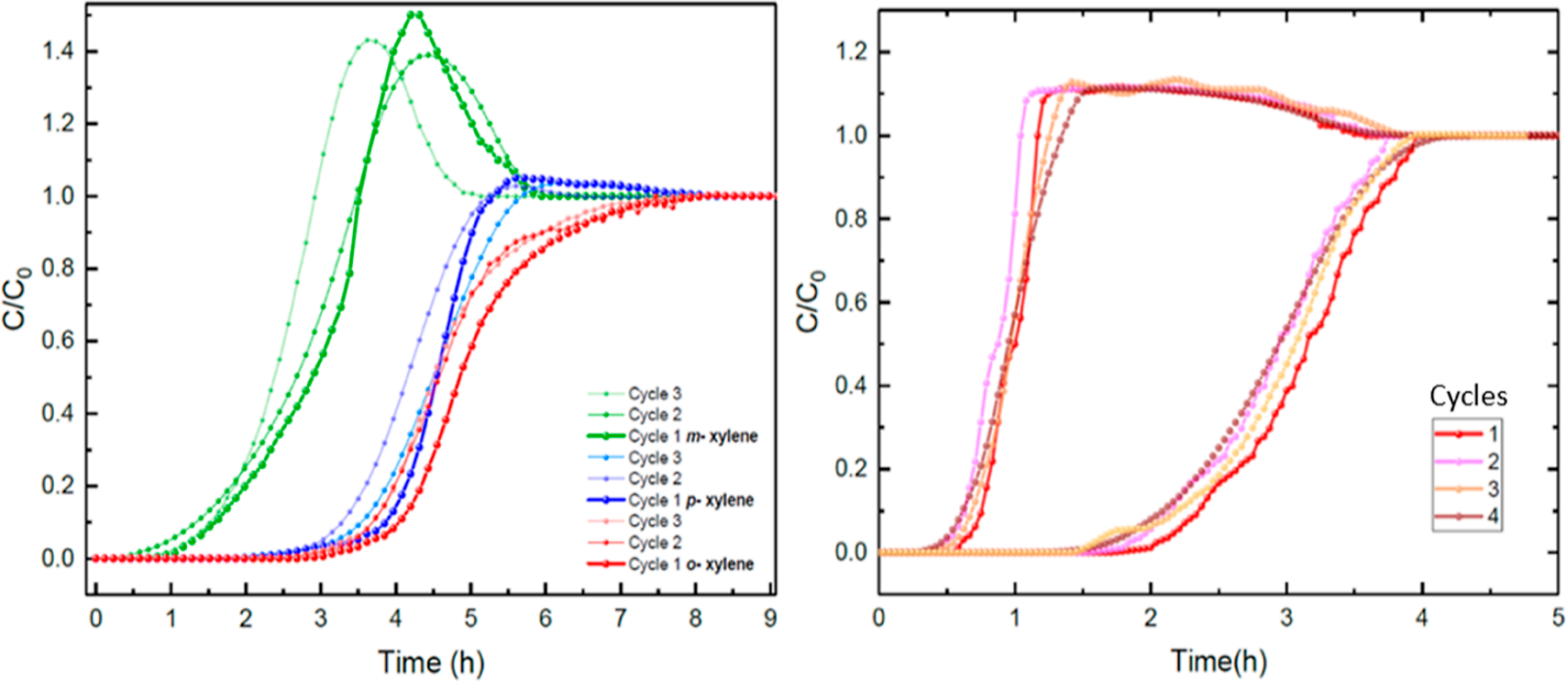
Reproducibility test of BCs of an equimolar gas mixture of xylene isomers *(left)* and a binary gas mixture of *n*-hexane/2,3-dimethylbutane (0.03%/0.04%, v/v) *(right).* Experiments from Figures 7 and 7, respectively.

The reusability of TAMOF-1 (Figure 15) was confirmed comparing the BCs after several separation/regeneration steps. The concentration profile perfectly indicated no degradation of TAMOF-1 after successive adsorption/desorption cycles and complete performance recovering under the studied operation conditions. Finally, after performing all the measurements for this work, TAMOF-1 was removed from the fixed-bed column to verify its chemical stability. The comparison of powder X-ray diffraction data between the fresh and used TAMOF-1 (see Figure S9) shows high chemical stability and robustness. It is worth mentioning that all experiments in this article with a 0.7 g bed were obtained with the very same sample, without any further treatment in between experiments that described the desorption step passing through nitrogen gas at the working temperature.

## Conclusions

We evaluated the separation performance of the chiral TAMOF-1 for several mixtures of organic compounds. We found that although the chirality of the structure assists the separation of chiral molecules, the characteristic shape and size of its channels as well as the presence of a hydrophobic main channel and small hydrophilic pockets (close to the open metal site) make TAMOF-1 a good candidate for separating a wide variety of achiral molecules as well. In particular, TAMOF-1 can separate xylene isomers, benzene from cyclohexane, and hexane isomers. The separation of the cyclic and aromatic molecules is based on the type of isomer or interaction with the metallic center (benzene–cyclohexane). The separation of xylene isomers is related to the interaction between the CH_3_ groups of the molecules and the ligands of the framework, while benzene–cyclohexane separation is related to the stronger interaction of the aromatic ring of the molecules of benzene. Moreover, TAMOF-1 can separate the linear and branched isotherms of hexane in a similar way to MFI zeolite. Since it has been reported that the crystallization method is useful to separate *o*-xylene,^62^ in a ternary mixture, with the combination of these two methods, the total separation of xylene isomers is possible.

Breakthrough experiments also confirm the computational results and show a great separation in the liquid and gas phases. TAMOF-1, an MOF with high thermal and water stability, is an excellent candidate for carrying out VOC separation and offers the possibility of regenerating and reusing it.

## Supporting Information Available

Complementary results including the snapshot of the structure of TAMOF-1; μVT MC simulations for the multi-component adsorption isotherms to validate IAST prediction; number of π-bond interactions per molecule for *o*-, *m*-, and *p*-xylene; single- and multi-component adsorption isotherms and adsorption selectivity of monobranched and dibranched hexane isomers in TAMOF-1 at 433 K; average occupation profile of *n*-hexane, dibranched isomers (2,3-dimethylbutane, 2,2-dimethylbutane), and monobranched isomers (3-methylpentane and 2-methylpentane) in TAMOF-1 at 433 K; heat of adsorption as a function of loading for the hexane isomers in TAMOF-1 at 433 K; number of π-bond interactions per molecule as a function of the adsorption loading in TAMOF-1 at 298 K; powder X-ray diffraction data of TAMOF-1 before and after the breakthrough experiments; RDFs between the CH_3_ groups of *o*-, *m*-, and *p*-xylene and the O atoms of the framework of the carboxylate groups of the framework; snapshot of the carboxylate group of the framework; RDFs between the CH_3_ groups of *o*-, *m*-, and *p*-xylene and the N4 and N5 atoms of the framework of the heterocycles of the framework; RDFs between the CH_3_ groups of *o*-, *m*-, and *p*-xylene and the N1, N2, and N3 atoms of the heterocycles of the framework; RDFs between the CH_3_ groups of hexane isomers and N2, N3, and N4 atoms; and RDFs between the C atoms of benzene and cyclohexane and the metal atoms of the framework (PDF)

## References

[ref1] Kang Y.-S.; Lu Y.; Chen K.; Zhao Y.; Wang P.; Sun W.-Y. Metal–Organic Frameworks with Catalytic Centers: From Synthesis to Catalytic Application. Coord. Chem. Rev. 2019, 378, 262–280. 10.1016/j.ccr.2018.02.009.

[ref2] Li B.; Wen H.-M.; Yu Y.; Cui Y.; Zhou W.; Chen B.; Qian G. Nanospace within Metal–Organic Frameworks for Gas Storage and Separation. Mater. Today Nano 2018, 2, 21–49. 10.1016/j.mtnano.2018.09.003. PMC11194750 38915818

[ref3] Ray Chowdhuri A.; Bhattacharya D.; Sahu S. K. Magnetic Nanoscale Metal Organic Frameworks for Potential Targeted Anticancer Drug Delivery, Imaging and as an MRI Contrast Agent. Dalton Trans. 2016, 45, 2963–2973. 10.1039/c5dt03736k. 26754449

[ref4] He Y.; Krishna R.; Chen B. Metal-Organic Frameworks with Potential for Energy-Efficient Adsorptive Separation of Light Hydrocarbons. Energy Environ. Sci. 2012, 5, 9107–9120. 10.1039/c2ee22858k.

[ref5] Cadiau A.; Adil K.; Bhatt P. M.; Belmabkhout Y.; Eddaoudi M. A Metal-Organic Framework – Based Splitter for Separating Propylene from Propane. Science 2016, 353, 137–140. 10.1126/science.aaf6323. 27387945

[ref6] Nugent P.; Belmabkhout Y.; Burd S. D.; Cairns A. J.; Luebke R.; Forrest K.; Pham T.; Ma S.; Space B.; Wojtas L.; Eddaoudi M.; Zaworotko M. J. Porous Materials with Optimal Adsorption Thermodynamics and Kinetics for CO_2_ Separation. Nature 2013, 495, 80–84. 10.1038/nature11893. 23446349

[ref7] Herm Z. R.; Bloch E. D.; Long J. R. Hydrocarbon Separations in Metal-Organic Frameworks. Chem. Mater. 2014, 26, 323–338. 10.1021/cm402897c.

[ref8] Verma P.; Xu X.; Truhlar D. G. Adsorption on Fe-MOF-74 for C1-C3 Hydrocarbon Separation. J. Phys. Chem. C 2013, 117, 12648–12660. 10.1021/jp402884h.

[ref9] Warren J. E.; Perkins C. G.; Jelfs K. E.; Boldrin P.; Chater P. A.; Miller G. J.; Manning T. D.; Briggs M. E.; Stylianou K. C.; Claridge J. B.; Rosseinsky M. J. Shape Selectivity by Guest-Driven Restructuring of a Porous Material. Angew. Chem., Int. Ed. 2014, 53, 4592–4596. 10.1002/anie.201307656. PMC4499242 24677281

[ref10] Corella-Ochoa M. N.; Tapia J. B.; Rubin H. N.; Lillo V.; González-Cobos J.; Núñez-Rico J. L.; Balestra S. R. G.; Almora-Barrios N.; Lledós M.; Güell-Bara A.; Cabezas-Giménez J.; Escudero-Adán E. C.; Vidal-Ferran A.; Calero S.; Reynolds M.; Martí-Gastaldo C.; Galán-Mascarós J. R. Homochiral Metal-Organic Frameworks for Enantioselective Separations in Liquid Chromatography. J. Am. Chem. Soc. 2019, 141, 14306–14316. 10.1021/jacs.9b06500. 31426632

[ref11] Niaz K.; Bahadar H.; Maqbool F.; Abdollahi M. Review Article: A Review of Environmental and Occupational Pharmaceutical Sciences Research Center, Tehran University of Medical Sciences, Health Authorities in Most Countries, like in Lene As100 Ppm in Industrial Place Where Xy- Ted to the Environment. EXCLI J. 2015, 14, 1167–1186. 10.17179/excli2015-623. 26862322 PMC4743476

[ref12] Cannella W. J. Xylenes and Ethylbenzene. Kirk–Othmer Encyclopedia of Chemical Technology; John Wiley & Sons, Ltd, 2000; pp 10–25.

[ref13] Krishna R. Separating Mixtures by Exploiting Molecular Packing Effects in Microporous Materials. Phys. Chem. Chem. Phys. 2015, 17, 39–59. 10.1039/c4cp03939d. 25377790

[ref14] Castillo J. M.; Vlugt T. J. H.; Calero S. Molecular Simulation Study on the Separation of Xylene Isomers in MIL-47 Metal-Organic Frameworks. J. Phys. Chem. C 2009, 113, 20869–20874. 10.1021/jp908247w.

[ref15] Peralta D.; Chaplais G.; Paillaud J.-L.; Simon-Masseron A.; Barthelet K.; Pirngruber G. D. The Separation of Xylene Isomers by ZIF-8: A Demonstration of the Extraordinary Flexibility of the ZIF-8 Framework. Microporous Mesoporous Mater. 2013, 173, 1–5. 10.1016/j.micromeso.2013.01.012.

[ref16] Jin Z.; Zhao H.-Y.; Zhao X.-J.; Fang Q.-R.; Long J. R.; Zhu G.-S. A Novel Microporous MOF with the Capability of Selective Adsorption of Xylenes. Chem. Commun. 2010, 46, 8612–8614. 10.1039/c0cc01031f. 20890493

[ref17] Rasouli M.; Yaghobi N.; Chitsazan S.; Sayyar M. H. Influence of Monovalent Cations Ion-Exchange on Zeolite ZSM-5 in Separation of Para-Xylene from Xylene Mixture. Microporous Mesoporous Mater. 2012, 150, 47–54. 10.1016/j.micromeso.2011.09.013.

[ref18] Rasouli M.; Yaghobi N.; Chitsazan S.; Sayyar M. H. Effect of Nanocrystalline Zeolite Na-Y on Meta-Xylene Separation. Microporous Mesoporous Mater. 2012, 152, 141–147. 10.1016/j.micromeso.2011.11.045.

[ref19] Khabzina Y.; Laroche C.; Perez-Pellitero J.; Farrusseng D. Xylene Separation on a Diverse Library of Exchanged Faujasite Zeolites. Microporous Mesoporous Mater. 2017, 247, 52–59. 10.1016/j.micromeso.2017.03.026.

[ref20] Yuan W.; Lin Y. S.; Yang W. Molecular Sieving MFI-Type Zeolite Membranes for Pervaporation Separation of Xylene Isomers. J. Am. Chem. Soc. 2004, 126, 4776–4777. 10.1021/ja031653t. 15080671

[ref21] Liu A.; Peng X.; Jin Q.; Jain S. K.; Vicent-Luna J. M.; Calero S.; Zhao D. Adsorption and Diffusion of Benzene in Mg-MOF-74 with Open Metal Sites. ACS Appl. Mater. Interfaces 2019, 11, 4686–4700. 10.1021/acsami.8b20447. 30618234

[ref22] Tu M.; Wannapaiboon S.; Khaletskaya K.; Fischer R. A. Engineering Zeolitic-Imidazolate Framework (ZIF) Thin Film Devices for Selective Detection of Volatile Organic Compounds. Adv. Funct. Mater. 2015, 25, 4470–4479. 10.1002/adfm.201500760.

[ref23] Mukherjee S.; Manna B.; Desai A. V.; Yin Y.; Krishna R.; Babarao R.; Ghosh S. K. Harnessing Lewis Acidic Open Metal Sites of Metal-Organic Frameworks: Foremost Route to Achieve Highly Selective Benzene Sorption over Cyclohexane. Chem. Commun. 2016, 52, 8215–8218. 10.1039/c6cc03015g. 27188914

[ref24] Jeong B. H.; Hasegawa Y.; Sotowa K. I.; Kusakabe K.; Morooka S. Permeation of Binary Mixtures of Benzene and Saturated C4-C7 Hydrocarbons through an FAU-Type Zeolite Membrane. J. Membr. Sci. 2003, 213, 115–124. 10.1016/s0376-7388(02)00518-5.

[ref25] Kobayashi Y.; Takami S.; Kubo M.; Miyamoto A. Computational Chemical Study on Separation of Benzene and Cyclohexane by a NaY Zeolite Membrane. Desalination 2002, 147, 339–344. 10.1016/s0011-9164(02)00606-9.

[ref26] Yang R. T.; Kikkinides E. S. New Sorbents for Olefin/Paraffin Separations via Adsorption via Pi-Complexation. AIChE J. 1995, 41, 509–517. 10.1002/aic.690410309.

[ref27] Luna-Triguero A.; Slawek A.; Sánchez-de-Armas R.; Gutiérrez-Sevillano J. J.; Ania C. O.; Parra J. B.; Vicent-Luna J. M.; Calero S. C. π-Complexation for Olefin-Paraffin Separation Using Aluminosilicates. Chem. Eng. J. 2020, 380, 1224482 10.1016/j.cej.2019.122482.

[ref28] Zhao J.; Sui P.; Wu B.; Chen A.; Lu Y.; Hou F.; Cheng X.; Cui S.; Song J.; Huang G.; Xing C.; Wang Q.-f. Benzene Induces Rapid Leukemic Transformation after Prolonged Hematotoxicity in a Murine Model. Leukemia 2021, 35, 595–600. 10.1038/s41375-020-0894-x. 32503976 PMC9205364

[ref29] Huff J. Benzene-Induced Cancers: Abridged History and Occupational Health Impact. Int. J. Occup. Environ. Health 2007, 13, 213–221. 10.1179/oeh.2007.13.2.213. 17718179 PMC3363002

[ref30] Glass D. C.; Gray C. N.; Jolley D. J.; Gibbons C.; Sim M. R.; Fritschi L.; Adams G. G.; Bisby J. A.; Manuell R. Leukemia Risk Associated with Low-Level Benzene Exposure. Epidemiology 2003, 14, 569–577. 10.1097/01.ede.0000082001.05563.e0. 14501272

[ref31] Dandekar H. W.; Funk G. A.; Zinnen H. A. Process for Separating and Recovering Multimethyl-Branched Alkanes. U.S. Patent 6,069,289 A, 2000.

[ref32] Dohner B. R.; John S.; Roby S. H.; Supp J. A. Process for Alkane Isomerization Using Reactive Chromatography. U.S. Patent 5,744,684 A, 1998.

[ref33] Krishna R.; van Baten J. M. Screening of Zeolite Adsorbents for Separation of Hexane Isomers: A Molecular Simulation Study. Sep. Purif. Technol. 2007, 55, 246–255. 10.1016/j.seppur.2006.12.011.

[ref34] Herm Z. R.; Wiers B. M.; Mason J. A.; Van Baten J. M.; Hudson M. R.; Zajdel P.; Brown C. M.; Masciocchi N.; Krishna R.; Long J. R. Separation of Hexane Isomers in a Metal-Organic Framework with Triangular Channels. Science 2013, 340, 960–964. 10.1126/science.1234071. 23704568

[ref35] Mendes P. A. P.; Rodrigues A. E.; Horcajada P.; Serre C.; Silva J. A. C. Single and Multicomponent Adsorption of Hexane Isomers in the Microporous ZIF-8. Microporous Mesoporous Mater. 2014, 194, 146–156. 10.1016/j.micromeso.2014.04.009.

[ref36] Bárcia P. S.; Ferreira A.; Gascon J.; Aguado S.; Silva J. A. C.; Rodrigues A. E.; Kapteijn F. Zeolite Beta Membranes for the Separation of Hexane Isomers. Microporous Mesoporous Mater. 2010, 128, 194–202. 10.1016/j.micromeso.2009.08.023.

[ref37] Mendes P. A. P.; Horcajada P.; Rives S.; Ren H.; Rodrigues A. E.; Devic T.; Magnier E.; Trens P.; Jobic H.; Ollivier J.; Maurin G.; Serre C.; Silva J. A. C. A Complete Separation of Hexane Isomers by a Functionalized Flexible Metal Organic Framework. Adv. Funct. Mater. 2014, 24, 7666–7673. 10.1002/adfm.201401974.

[ref38] Ferreira A. F. P.; Mittelmeijer-Hazeleger M. C.; Bergh J. V. D.; Aguado S.; Jansen J. C.; Rothenberg G.; Rodrigues A. E.; Kapteijn F. Adsorption of Hexane Isomers on MFI Type Zeolites at Ambient Temperature: Understanding the Aluminium Content Effect. Microporous Mesoporous Mater. 2013, 170, 26–35. 10.1016/j.micromeso.2012.11.020.

[ref39] Ferreira A. F. P.; Mittelmeijer-Hazeleger M. C.; Bliek A. Can Alkane Isomers Be Separated? Adsorption Equilibrium and Kinetic Data for Hexane Isomers and Their Binary Mixtures on MFI. Adsorption 2007, 13, 105–114. 10.1007/s10450-007-9010-z.

[ref40] Couri D.; Milks M. Toxicity and Metabolism of the Neurotoxic Hexacarbons N-Hexane, 2-Hexanone, and 2, 5-Hexanedione. Annu. Rev. Pharmacol. Toxicol. 1982, 22, 145 10.1146/annurev.pa.22.040182.001045. 7044283

[ref41] Takeuchi Y.; Ono Y.; Hisanaga N.; Kitoh J.; Sugiura Y. A Comparative Study of the Toxicity of N-Pentane, n-Hexane, and n-Heptane to the Peripheral Nerve of the Rat. Clin. Toxicol. 1981, 18, 1395–1402. 10.3109/15563658108990348. 6277550

[ref42] Dubbeldam D.; Calero S.; Ellis D. E.; Snurr R. Q. RASPA: Molecular Simulation Software for Adsorption and Diffusion in Flexible Nanoporous Materials. Mol. Simul. 2016, 42, 81–101. 10.1080/08927022.2015.1010082.

[ref43] Wilmer C. E.; Kim K. C.; Snurr R. Q. An Extended Charge Equilibration Method. J. Phys. Chem. Lett. 2012, 3, 2506–2511. 10.1021/jz3008485. 26292141

[ref44] Buchholz R.; Kraetzer C.; Dittmann J. UFF, a Full Periodic Table Force Field for Molecular Mechanics and Molecuar Dynamics Simulations. Lecture Notes in Computer Science (including subseries Lecture Notes in Artificial Intelligence and Lecture Notes in Bioinformatics), 2009; Vol. 5806 LNCS, pp 235–246.

[ref45] Mayo S. L.; Olafson B. D.; Goddard W. A. DREIDING: A Generic Force Field for Molecular Simulations. J. Phys. Chem. 1990, 94, 8897–8909. 10.1021/j100389a010.

[ref46] Yazaydin A. Ö.; Benin A. I.; Faheem S. A.; Jakubczak P.; Low J. J.; Richard R. W.; Snurr R. Q. Enhanced CO2 Adsorption in Metal-Organic Frameworks via Occupation of Open-Metal Sites by Coordinated Water Molecules. Chem. Mater. 2009, 21, 1425–1430. 10.1021/cm900049x.

[ref47] Karra J. R.; Walton K. S. Effect of Open Metal Sites on Adsorption of Polar and Nonpolar Molecules in Metal–Organic Framework Cu-BTC. Langmuir 2008, 24, 8620–8626. 10.1021/la800803w. 18630977

[ref48] Vicent-Luna J. M.; Gutiérrez-Sevillano J. J.; Hamad S.; Anta J.; Calero S. Role of Ionic Liquid [EMIM]+[SCN]– in the Adsorption and Diffusion of Gases in Metal–Organic Frameworks. Chem. Eng. J. 2018, 10, 29694–29704. 10.1021/acsami.8b11842. 30089205

[ref49] Calero S.; Gómez-Álvarez P. Underlying Adsorption Mechanisms of Water in Hydrophobic and Hydrophilic Zeolite Imidazolate Frameworks: ZIF-71 and ZIF-90. J. Phys. Chem. C 2015, 119, 23774–23780. 10.1021/acs.jpcc.5b07360.

[ref50] Rai N.; Siepmann J. I. Transferable Potentials for Phase Equilibria. 10. Explicit-Hydrogen Description of Substituted Benzenes and Polycyclic Aromatic Compounds. J. Phys. Chem. B 2013, 117, 273–288. 10.1021/jp307328x. 23205778

[ref51] Widom B. Some Topics in the Theory of Fluids. J. Chem. Phys. 1963, 39, 2808–2812. 10.1063/1.1734110.

[ref52] Bell L. N. Thermodynamics of Mixed-Gas Adsorption. Biophysics 1965, 9, 316–321.

[ref53] Balestra S. R. G.; Bueno-Perez R.; Calero S. GAIAST. Zenodo DataSet 2016, 10.5281/zenodo.596674.

[ref54] Krishna R.; van Baten J. M. How Reliable Is the Ideal Adsorbed Solution Theory for the Estimation of Mixture Separation Selectivities in Microporous Crystalline Adsorbents?. ACS Omega 2021, 6, 15499–15513. 10.1021/acsomega.1c02136. 34151128 PMC8210411

[ref55] Martinez C. R.; Iverson B. L. Rethinking the Term “Pi-Stacking. Chem. Sci. 2012, 3, 2191–2201. 10.1039/c2sc20045g.

[ref56] Dance I. Distance Criteria for Crystal Packing Analysis of Supramolecular Motifs. New J. Chem. 2003, 27, 22–27. 10.1039/b206867b.

[ref57] He C.-T.; Jiang L.; Ye Z.-M.; Krishna R.; Zhong Z.-S.; Liao P.-Q.; Xu J.; Ouyang G.; Zhang J.-P.; Chen X.-M.; Moe †. Exceptional Hydrophobicity of a Large-Pore Metal-Organic Zeolite. J. Am. Chem. Soc. 2015, 137, 7217–7223. 10.1021/jacs.5b03727. 25985194

[ref58] Münch A. S.; Mertens F. O. R. L. HKUST-1 as an Open Metal Site Gas Chromatographic Stationary Phase - Capillary Preparation, Separation of Small Hydrocarbons and Electron Donating Compounds, Determination of Thermodynamic Data. J. Mater. Chem. 2012, 22, 10228–10234. 10.1039/c2jm15596f.

[ref59] Yang Y.; Bai P.; Guo X. Separation of Xylene Isomers: A Review of Recent Advances in Materials. Ind. Eng. Chem. Res. 2017, 56, 14725–14753. 10.1021/acs.iecr.7b03127.

[ref60] de Malsche W.; van der Perre S.; Silverans S.; Maes M.; de Vos D. E.; Lynen F.; Denayer J. F. M. Unusual Pressure-Temperature Dependency in the Capillary Liquid Chromatographic Separation of C8 Alkylaromatics on the MIL-53(Al) Metal-Organic Framework. Microporous Mesoporous Mater. 2012, 162, 1–5. 10.1016/j.micromeso.2012.06.002.

[ref61] Bozbiyik B.; Duerinck T.; Lannoeye J.; de Vos D. E.; Baron G. v.; Denayer J. F. M. Adsorption and Separation of N-Hexane and Cyclohexane on the UiO-66 Metal-Organic Framework. Microporous Mesoporous Mater. 2014, 183, 143–149. 10.1016/j.micromeso.2013.07.035.

[ref62] Weedman J. A. Separation of Xylene Isomers by Cristallization and Distillation. U.S. Patent 3,067,270 A, 1962.

