# Supplementary material for: Separation of Volatile Organic Compounds in TAMOF-1
Source: ACS Appl Mater Interfaces. 2022 Jul 1;14(27):30772–85. doi: 10.1021/acsami.2c05223 (PMC9679997; doi:10.1021/acsami.2c05223)
Supplement: Supplementary file 1 — am2c05223_si_001.pdf [file am2c05223_si_001.pdf]

Supporting Information for:
Separation of Volatile Organic Compounds in TAMOF-1

Carmen González-Galán,[a,#] Mabel de Fez-Febré,[b,c,#] Stefano Giancola,[b] Jesús González-Cobos,[b,†] Anton Vidal-Ferran,[d,g] José Ramón Galán-Mascarós,[b,d] Salvador R. G. Balestra,[a,e,*] and Sofía Calero [a,f,*]*

[a)] Department of Physical, Chemical, and Natural Systems, Universidad Pablo de Olavide. Ctra. Utrera km 1. ES-41013 Seville, Spain

[b)] Institute of Chemical Research of Catalonia (ICIQ), The Barcelona Institute of Science and Technology (BIST), Av. Països Catalans 16. ES-43007 Tarragona, Spain

[c)] Departament de Química Física i Inorgànica, Universitat Rovira i Virgili, Marcel. lí Domingo 1, 43007 Tarragona, Spain.

[d)] Catalan Institution for Research and Advanced Studies (ICREA), Passeig Lluis Companys 23. ES-08010 Barcelona, Spain

[e)] Instituto de Ciencia de Materiales de Madrid, Consejo Superior de Investigaciones Científicas (ICMM-CSIC), c/ Sor Juana Inés de la Cruz, 3, 28049 Madrid, Spain

[f)] Materials Simulation and Modelling, Department of Applied Physics, Eindhoven University of Technology, 5600 MB Eindhoven, The Netherlands

[g)] Department of Inorganic and Organic Chemistry, University of Barcelona, C. Martí i Franquès 1-11, 08028 Barcelona, Spain

[#] These authors contributed equally to the manuscript

Author Information

José Ramón Galán-Mascarós (e-mail: jrgalan@iciq.es)

Salvador R. G. Balestra (e-mail: salrodgom@upo.es)

Sofía Calero (e-mail: s.calero@tue.nl)

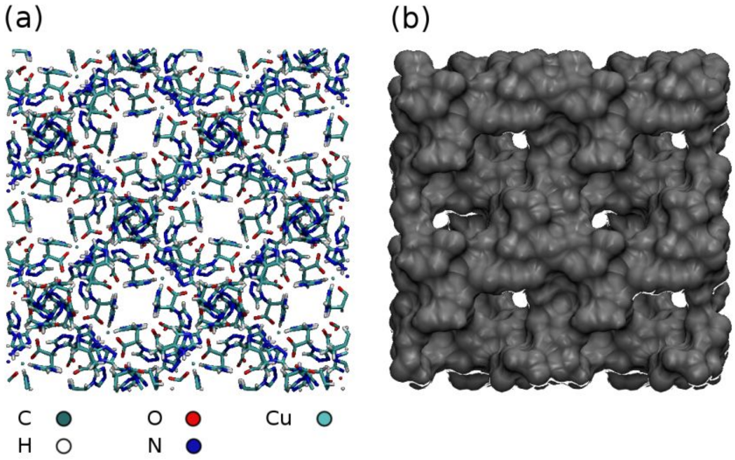

Figure S1. Snapshot of the structure of TAMOF-1 (C atoms are represented in green, H in white, O in red, N in purple).

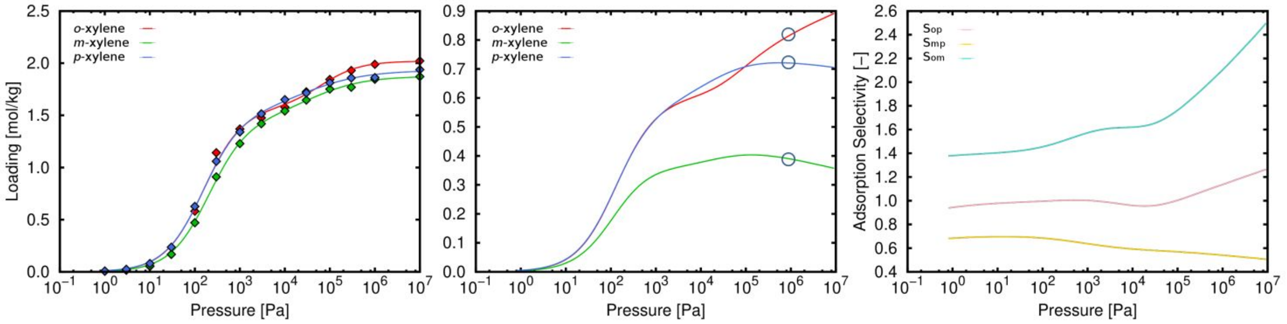

Figure S2. μVT MC simulations (empty symbols) for the multi-component adsorption isotherms to validate IAST prediction.

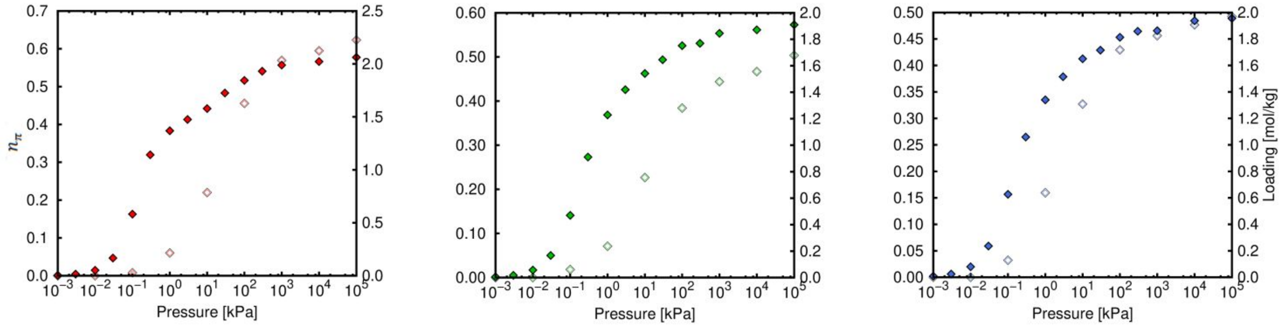

Figure S3. Number of π-bond interactions per molecule ($n_\pi = N_\pi M^{-1}$) for (a) *o*-xylene, (b) *m*-xylene and (c) *p*-xylene. Full symbols and empty symbols are related to the n_π and the single adsorption isotherms at 393 K, respectively.

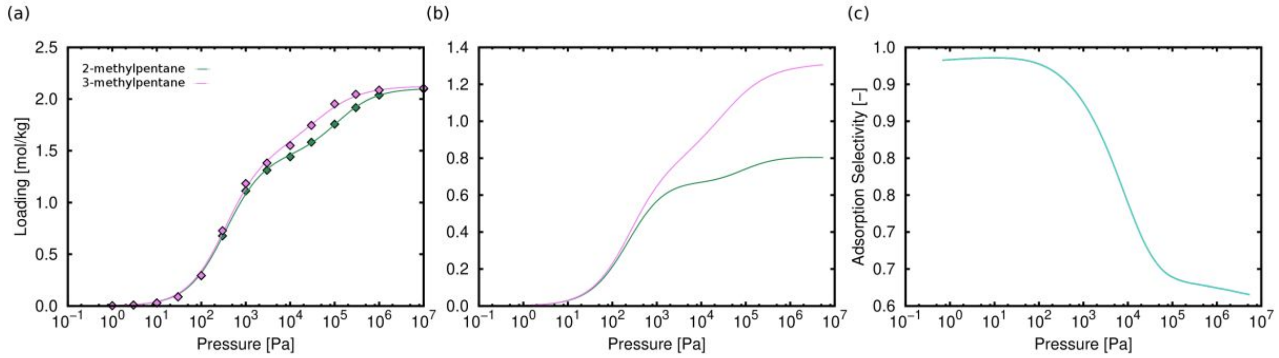

Figure S4. (a) Single and (b) multi-component adsorption isotherms and (c) adsorption selectivity of the monobranched hexane isomers in TAMOF-1 at 433 K.

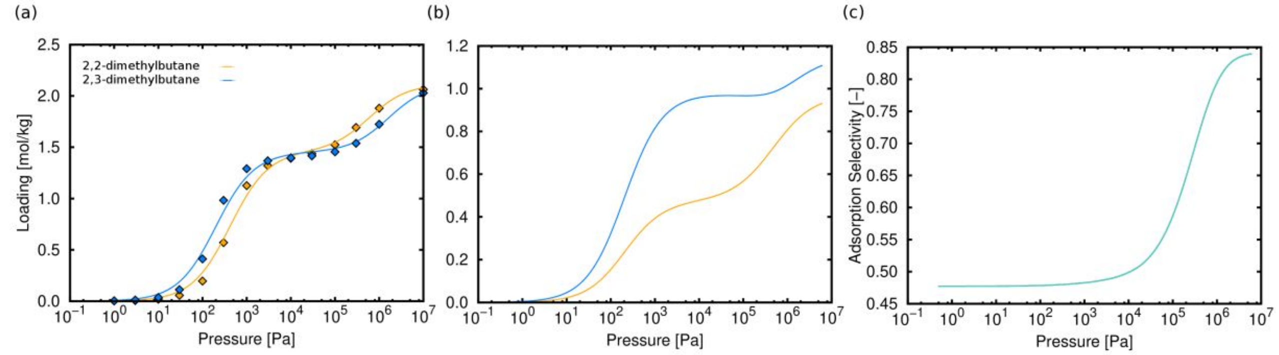

Figure S5. (a) Single and (b) multi-component adsorption isotherms and (c) adsorption selectivity of the dibranched hexane isomers in TAMOF-1 at 433 K.

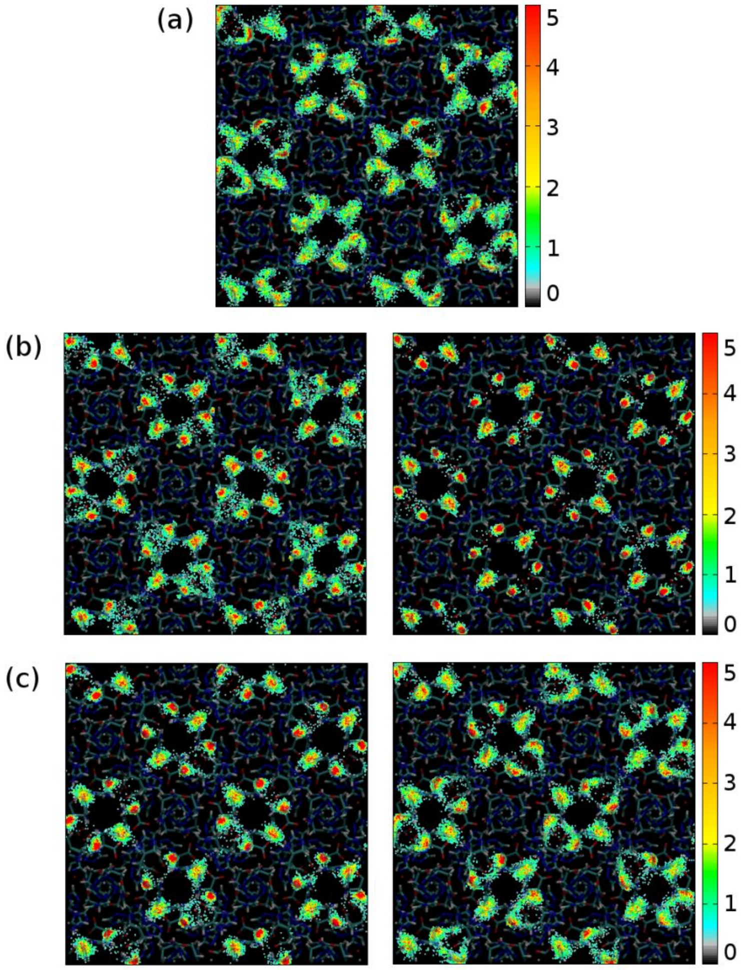

Figure S6. Average occupation profile of (a) *n*-hexane, (b) dibranched isomers, 2,3-dimethylbutane (left) and 2,2-dimethylbutane (right) and (c) monobranched isomers, 3-methylpentane (left) and 2-methylpentane (right) in TAMOF-1 at 433 K.

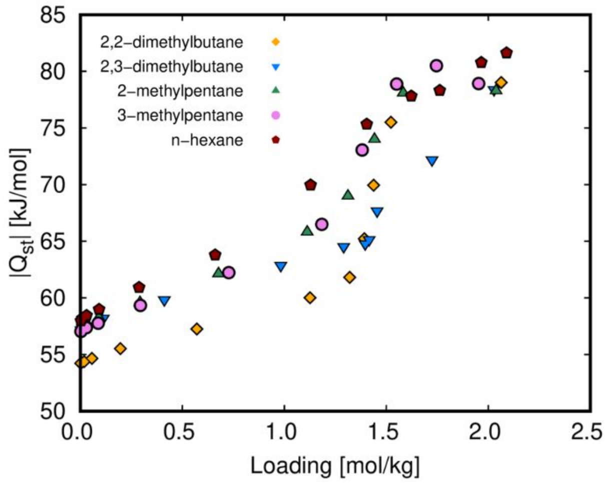

Figure S7. Heat of adsorption as a function of loading for the hexane isomers in TAMOF-1 at 433 K.

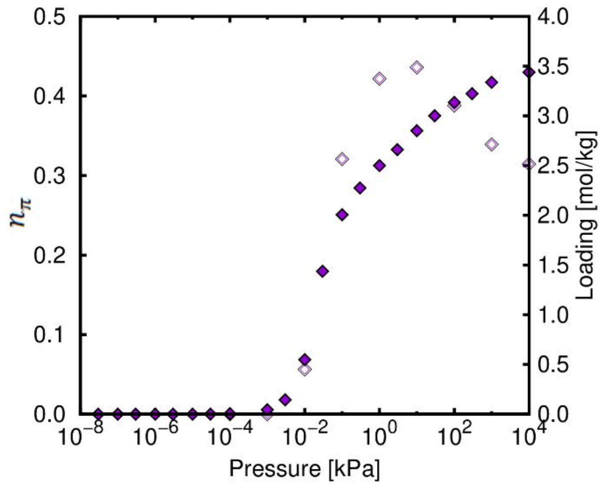

Figure S8. Number of π-bond interactions per molecule (full symbols) as a function of the adsorption loading (empty symbols) in TAMOF-1 at 298 K.

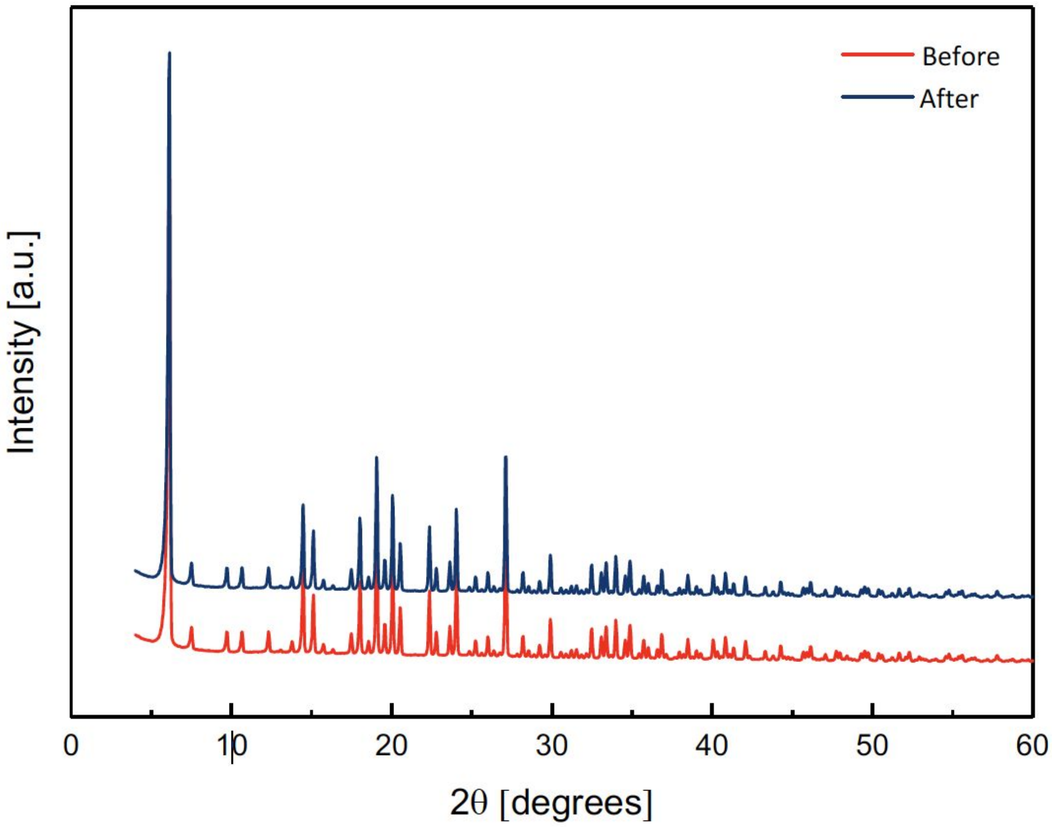

Figure S9. Powder X-Ray diffraction (PXRD) data of TAMOF-1 before (red) and after (blue) the breakthrough experiments.

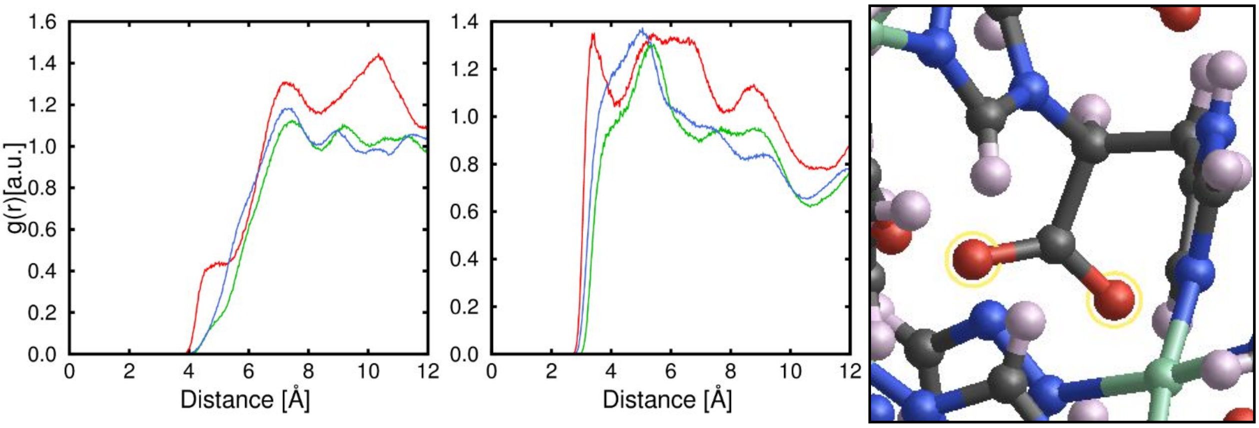

Figure S10. (**Left**) Radial distribution functions between the CH_3 groups of *o*-, *m*-, and *p*-xylene and the O atoms of the framework (O_1 and O_2) of the carboxylate groups of the framework. (**Right**) Snapshot of the carboxylate group of the framework. O_2 is more exposed in the pore. The code color is red, green, and blue for *o*-, *m*- and *p*-xylene, respectively.

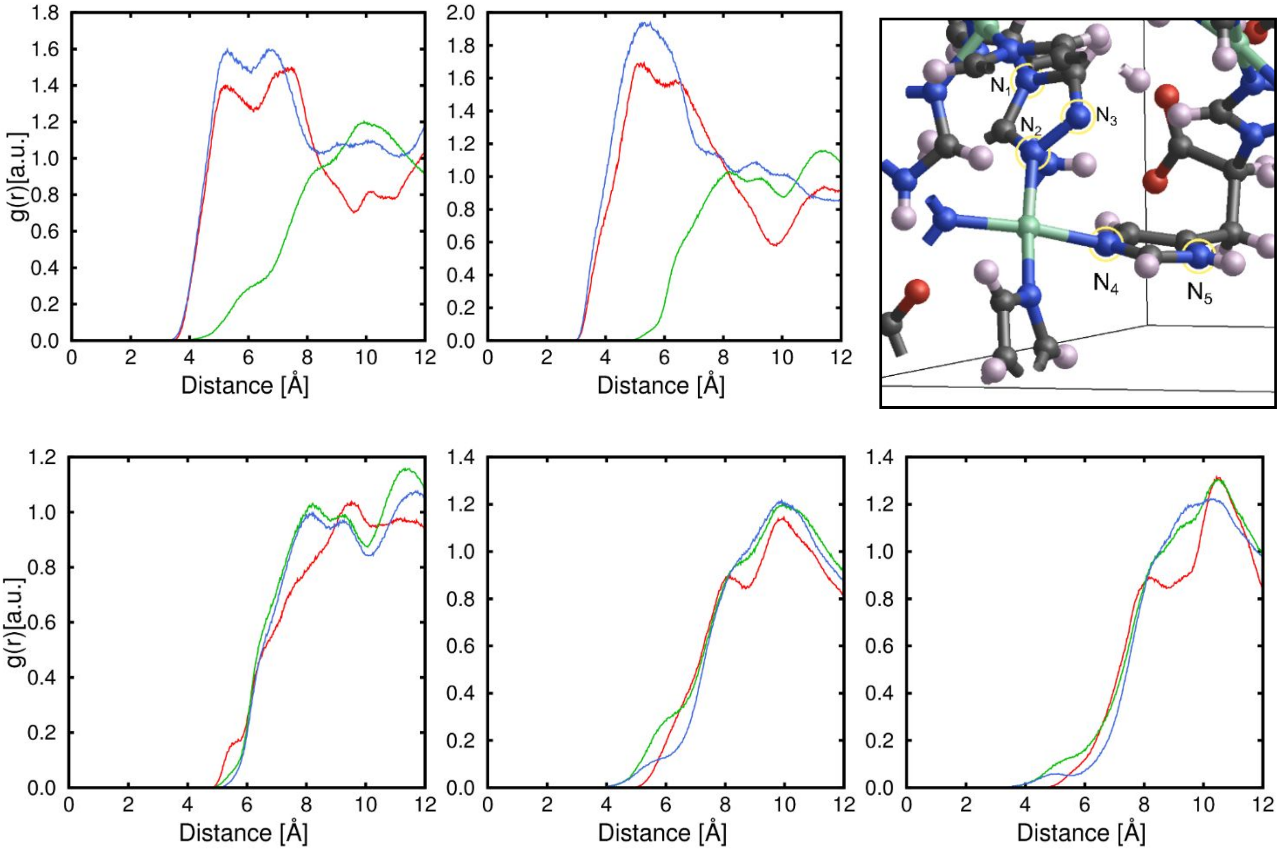

Figure S11. (**Top-Left**) Radial distribution functions between the CH_3 groups of *o*-, *m*-, and *p*-xylene and the N_4-, and N_5-atoms of the framework of the heterocycles of the framework. These nitrogen atoms are more exposed in the pore. (**Bottom**) Radial distribution functions between the CH_3 groups of *o*-, *m*-, and *p*-xylene and the N_1-, N_2-, and N_3-atoms of the framework of the heterocycles of the framework. These nitrogen atoms are less exposed in the pore and RDFs add little to the interpretation of xylene separation. (**Top-Right**) Capture of the structure with the labels of the nitrogen atoms.

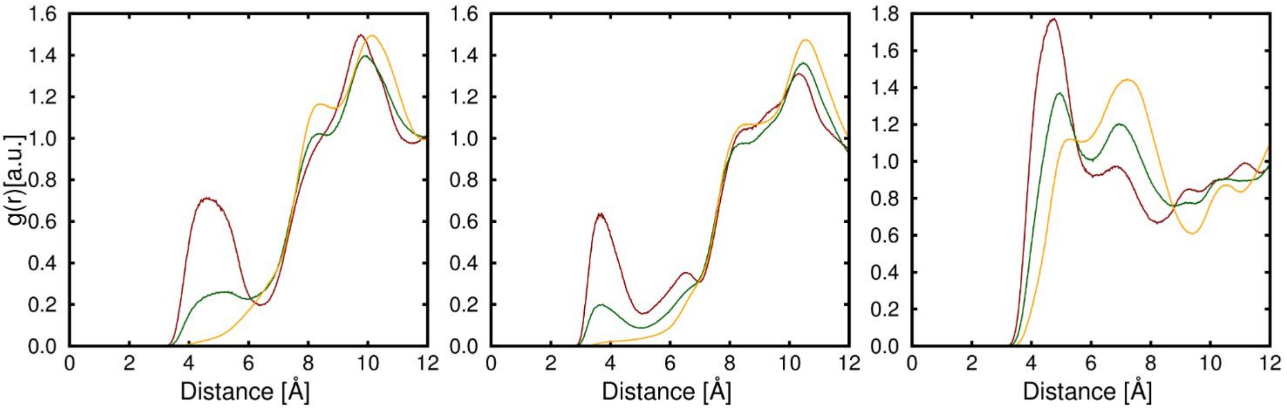

Figure S12. Radial distribution functions between the CH_3 groups of the hexane isomers (hexane in brown, monobranched isomer in green and dibranched isomer in yellow) and N_2, N_3 and N_4 (**from left to right**) of the framework. The position of N_2, N_3 and N_4 are shown in **Figure S11**.

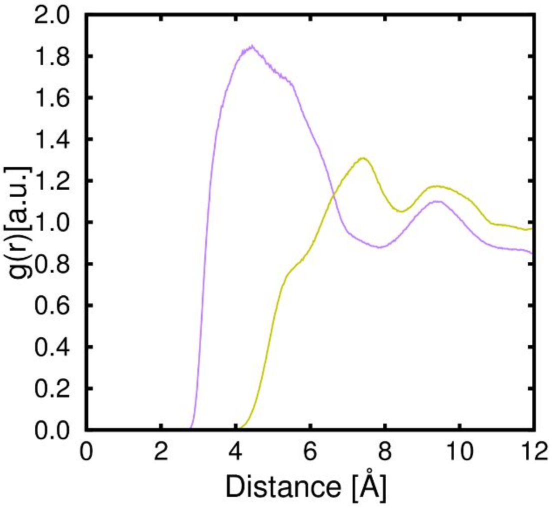

Figure S13. Radial distribution functions between the C atoms of benzene (purple line) and cyclohexane (yellow line) and the metal atoms of the framework.
